# Robots in Crisis and Disaster Management: Advances in Technology, Applications, and Emerging Trends

**DOI:** 10.3390/s26206518

**Published:** 2026-10-14

**Authors:** Grzegorz Wilk-Jakubowski, Daniel Kaczmarski, Jacek Wilk-Jakubowski

**Affiliations:** 1 Institute of Internal Security, Old Polish University of Applied Sciences, 49 Ponurego Piwnika Str., 25-666 Kielce, Poland; grzegorzwilkjakubowski@wp.pl; 2 Institute of Crisis Management and Computer Modelling, 28-100 Busko-Zdrój, Poland; 3 Department of Information Systems, Kielce University of Technology, 7 Tysiąclecia Państwa Polskiego Ave., 25-314 Kielce, Poland; d.kaczmarski@tu.kielce.pl

**Keywords:** crisis management, disaster management, disaster robotics, unmanned aerial vehicles, search and rescue, risk management, human–robot interaction, multi-robot systems, situational awareness, resource and knowledge management, engineering

## Abstract

Robotic systems can support crisis and disaster management when direct human intervention would be dangerous or delayed. This review examines 76 English-language publications indexed in Scopus and Web of Science and published from 2006 to July 2025. The studies were classified across three management domains and three Robotics Ecosystem categories. Within the reviewed sample, most publications appeared after 2015, and conference papers were the dominant document type. None of the seven reported chi-square statistics had a *p*-value below 0.05. Five comparisons involved overlapping labels and were therefore treated only as exploratory summaries; the descriptive counts and qualitative findings received greater interpretive weight. The reviewed literature suggests that no single robotic platform meets the demands of every disaster scenario. UAVs are useful for surveying, mapping, and observing inaccessible areas, while ground and specialized robots support close inspection, environmental sensing, and work in confined or unstable environments. Multi-robot systems can extend mission coverage but also increase the demands on communication, coordination, data fusion, task allocation, and operator supervision. Many of the included solutions were evaluated only in simulations, prototype demonstrations, or short experiments. Recurrent limitations concerned field validation, perception, localization, real-time processing, communication, autonomy, interoperability, reliability, energy use, and human–robot collaboration. Future studies should test complete systems in repeatable field exercises and retain human supervision over safety-critical decisions.

## 1. Introduction

A disaster rarely allows responders to develop a complete situational picture before emergency operations begin. Rescue personnel may have to work amid blocked roads, damaged power and telecommunications infrastructure, and incomplete information about hazards and affected people. Crisis and disaster management therefore extends beyond immediate response. It also encompasses risk reduction, preparedness, resource coordination, and the preservation of knowledge required during recovery and subsequent decision-making [1,2].

Technology alone cannot solve these problems. Information must reach decision-makers early enough to influence their actions, while the teams involved in an operation should have access to a reasonably consistent picture of the event. During emergency operations, communication, coordination, cognition, and control are closely interconnected. At the same time, the allocation of limited personnel, transportation capacity, sensors, equipment, and supplies directly affects the pace and effectiveness of humanitarian assistance [3,4].

Robotic systems can reduce the gap between the demand for information and the risks involved in obtaining it. They can enter an unstable building, measure contamination, inspect a landslide, or survey a large area before rescue personnel are deployed. Field experience also helps explain why a successful laboratory demonstration does not necessarily indicate operational readiness: mobility, communication, interface design, equipment maintenance, and cooperation with rescue personnel must all work at the same time [5,6,7,8,9]. The purpose of a robot is therefore not to replace a rescue team, but to extend its operational reach and provide information that would otherwise be delayed, incomplete, or unavailable.

No single platform is suitable for every emergency. Unmanned aerial vehicles (UAVs) can rapidly survey large areas, produce maps, and identify locations that require closer inspection. Ground robots can approach a source of danger, enter damaged structures, or carry sensors and supplies. Continuum, legged, modular, and soft robots address more specialized mobility requirements. Studies have described systems for hazardous-gas detection, confined-space inspection, integrated response networks, victim detection, wildfire response, and heterogeneous robotic teams designed to operate in landslide environments [10,11,12,13,14,15].

In practice, the challenge increasingly concerns the coordination of several devices rather than the selection of a single robot. An aerial platform may perform an initial survey, a ground robot may inspect a selected location, and a wireless sensor network (WSN) may continue monitoring after the mobile platforms have been withdrawn. Such arrangements increase system coverage and resilience, but they also create new requirements related to energy management, interoperability, security, and the maintenance of a common operational picture [12,16,17,18,19,20].

Mobility and perception represent only part of the problem. The shortest route may pass through unstable terrain, a smoke-filled building, or an area occupied by rescue personnel. A plan must be updated as conditions change, and a robot must respond safely to obstacles, sensor failures, or loss of communication. In human–robot interaction (HRI), predictable behavior, an appropriate level of autonomy, and a clear method of transferring control are as important as the mechanical performance of the platform [21,22,23,24,25,26,27].

Data acquisition is also only the first stage. Information must retain its meaning, provenance, and timeliness as it moves between robots, databases, and decision-makers. Distributed communication gateways, semantic knowledge networks, and graph-synchronization methods can reduce dependence on a single communication center. However, they do not automatically resolve inconsistencies between observations or determine which source should be trusted [18,28,29,30,31,32,33].

The available literature describes numerous robotic platforms, algorithms, interfaces, and communication systems. However, these solutions are evaluated much less frequently as parts of a complete management process. As a result, it is not always clear whether a proposed system primarily improves direct emergency response, reduces risk before or during a mission, or supports resource and knowledge management. A classification based exclusively on robot type would not capture these differences.

Previous studies provide valuable information on rescue robots, multi-robot systems, UAVs, and experience gained from real-world operations [6,7,8,9]. However, this knowledge remains dispersed across technical studies, reports on individual applications, and analyses of selected deployments. A combined review is therefore needed to show how the field has developed over the past two decades, which management functions have received the greatest attention, which technologies and research methods have dominated, and which problems continue to restrict operational use.

This study addresses these issues through a structured review of 76 unique English-language publications published between January 2006 and July 2025. The quantitative analysis was combined with a qualitative synthesis across three management domains: Crisis Management and Response, Risk Management and Planning, and Resource and Knowledge Management. A complementary, cross-cutting Robotics Ecosystem perspective covered General and Specialized Robots, Unmanned Aerial Vehicles, and Technologies and Applications. To organize the available evidence and connect the technological perspective with the requirements of crisis and disaster management, five research questions (RQs) were formulated:

1. RQ1: What trends in publication volume, document type, and geographic distribution can be identified in research on the use of robotics in crisis and disaster management between 2006 and 2025?
2. RQ2: Which management domains, Robotics Ecosystem categories, and research methodologies occur most frequently, and how did their prevalence change between 2006–2015 and 2016–2025?
3. RQ3: Are the Robotics Ecosystem categories and research methodologies associated with the dominant management domain?
4. RQ4: What conclusions can be drawn from the qualitative analysis regarding robot applications, operational readiness, and support for reconnaissance, planning, communication, and resource and knowledge management?
5. RQ5: Which limitations and research gaps recur most frequently, and which future research directions could bring robotic systems closer to reliable operational use?

The remainder of the paper is structured as follows. Section 2 describes the materials and methods, including the search procedure, study selection, classification framework, and analytical approach. Section 3 presents the results of the quantitative analysis of the 76-publication dataset. Section 4 provides a qualitative synthesis of the state of the art across the three management domains and the Robotics Ecosystem categories. Section 5 discusses the findings and examines the ten most frequently recurring limitations and research gaps. Finally, Section 6 summarizes the main conclusions and identifies priorities for future research.

## 2. Materials and Methods

This study was designed as a structured literature review that combines a quantitative description of the field with a qualitative synthesis of its relevance to crisis-management practice. The review covered publications issued from January 2006 through July 20, 2025. The studies examined different platforms, tasks, technologies, and outcomes rather than one narrowly defined intervention, so a pooled effect estimate would not have been appropriate. The analysis instead considered publication patterns, management tasks, research methods, operational capabilities, and the limitations reported in the selected studies.

The five questions introduced in Section 1 determined which information was entered in the worksheet and how the findings were subsequently organized. This section describes how the material was handled; the answers are presented in Section 3, Section 4, Section 5 and Section 6.

### 2.1. Review Design and Scope

To compare change over time, the study period was divided into 2006–2015 and 2016–2025. The boundary separates the earlier use of rescue robots from the period in which UAVs, multi-robot systems, intelligent sensors, and data-driven autonomy became more prominent. The unit of analysis was a single publication. The number of studies therefore remained 76 even when one publication received several labels.

A publication was eligible when it connected a robotic platform, a supporting technology, or a capability applicable to robotics with a recognizable task in crisis management, disaster management, rescue, risk, resources, information, the environment, or planning. A paper without a direct disaster scenario could remain in the set if it examined a capability clearly needed in crisis operations, such as mobility, stability, reconnaissance, HRI, communication, planning, or resource allocation. Each such decision was justified in the worksheet. Document type did not determine inclusion; thematic relevance and the availability of data needed for coding did.

The review distinguished directly relevant studies from enabling or peripheral studies. Directly relevant studies explicitly addressed a disaster, crisis, emergency, search-and-rescue, or responder setting. A study without such a scenario was retained only when the investigated capability could be linked to a specific operational requirement already defined for crisis work, such as movement over unstable terrain, sensing in poor visibility, communication after infrastructure loss, multi-robot coordination, human supervision, or operation under strict energy and payload limits. General robotics studies were excluded when the connection rested only on a possible future use and no identifiable crisis-management task or operating constraint could be assigned. This boundary was applied during full-text assessment and is also used to distinguish evidence about complete crisis systems from evidence about enabling components.

### 2.2. Literature Search and Publication Selection

Scopus and Web of Science (WoS) were searched during the same search period for English-language publications issued from 2006 through 2025. The same search logic, thematic concept groups, publication-year range, and language restriction were applied in both databases. Because Scopus and WoS use different field codes and query syntax, the equivalent strategy was expressed in a database-specific form. In Scopus, the TITLE-ABS field combined (“Crisis Management” OR “Disaster Management”) with “Robots”. The results were limited to the specified publication years, English, selected subject areas relevant to robotics and management, and document types other than letters. Management-related exact keywords included Disaster Management, Crisis Management, Human Resource Management, Risk Management, Environmental Management, Information Management, Search and Rescue, Simulation, Situation Awareness, Distributed Knowledge, and Motion Planning.

The first Scopus filtering stage returned 115 records. A second exact-keyword block was then applied for robotic platforms and enabling technologies, including Robots, Robotics, Intelligent Robots, Multipurpose Robots, Industrial Robots, Rescue Robot, Mobile Robot, Multi-robot Systems, Swarm Robotics, UAV and drone terms, Robot Programming, Robot Learning, Human Robot Interaction, Sensors, Cameras, Wireless Sensor Networks, the Internet of Things, and related applications. This reduced the set to 110 records. Nine records were then removed as outside the scope of the review, leaving 101 Scopus records for the subsequent combined selection process.

The WoS search formed part of the same search phase and followed the same conceptual query, thematic keyword groups, publication-year range, and English-language restriction as the Scopus search. The database-specific expression necessarily used WoS field tags and syntax rather than Scopus commands. The exact text originally entered in WoS was not retained, so the WoS expression was rebuilt from the retained export and documented search conditions. The initial WoS search returned 117 records. Applying the documented keyword refinement reduced this set to 29 records, so 88 records were not taken forward. These 29 records were merged with the 101 Scopus records, giving 130 records before deduplication. DOI matching followed by normalized-title matching identified 19 records present in both databases and one additional duplicate within Scopus. Removing these 20 duplicate records left 110 unique records for eligibility assessment.

The complete reconstructed WoS expression and its documented refinement blocks are provided in Appendix A. The appendix identifies the expression as a reconstruction from the retained export and search notes; it is not presented as the verbatim query originally entered in WoS.

Comparison of the 110 unique records with the retained final dataset showed that 34 records were not included and that 76 unique publications met the review criteria [10,11,12,13,14,15,16,17,18,19,20,21,22,23,24,25,26,27,28,29,30,31,32,33,34,35,36,37,38,39,40,41,42,43,44,45,46,47,48,49,50,51,52,53,54,55,56,57,58,59,60,61,62,63,64,65,66,67,68,69,70,71,72,73,74,75,76,77,78,79,80,81,82,83,84,85]. Some records were removed because the full text could not be obtained, whereas others were excluded after full-text assessment. The retained files did not preserve a separate reason code for every excluded record, so the total of 34 cannot be divided reliably between unavailable full texts and full-text eligibility exclusions. References [1,2,3,4,5,6,7,8,9] were used only for the conceptual and operational background and were not included in the counts, tables, figures, or statistical tests. 

In practice, the article features six summary tables. Table 1 classifies the literature by document type, management domain, robotics ecosystem, and methodology, while Table 2 tracks publication trends over time. Table 3 breaks down the publications by period and affiliation country. Table 4 illustrates how robotics ecosystems and methodologies intersect across dominant management domains. Finally, Table 5 compares the main platform and system classes, and Table 6 highlights the most common limitations and research gaps.

**Figure 1:**
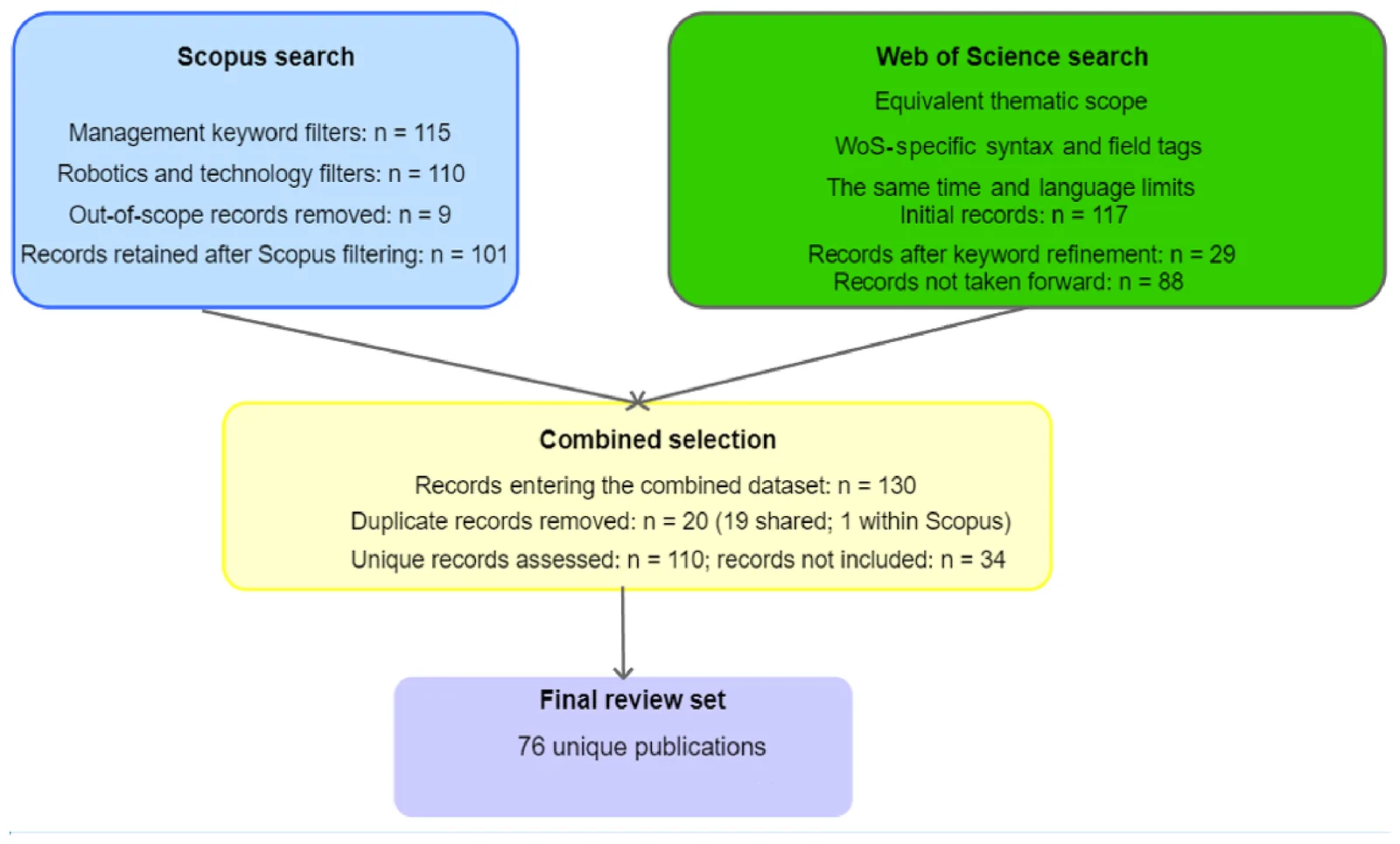
Database search, deduplication, and study selection process.

Figure 1 reports every stage that can be verified from the retained exports and notes. Scopus produced 115 records after the first thematic filter, 110 after the robotics and technology refinement, and 101 after nine out-of-scope records were removed. The equivalent WoS search, conducted during the same search period, produced 117 records, of which 29 remained after refinement. The combined set therefore contained 130 records. Removing 19 records shared by both databases and one duplicate within Scopus left 110 unique records for eligibility assessment. Of these, 76 were included and 34 were not included. The retained files do not contain a separate reason code for each of the 34 non-included records, so they cannot be divided reliably between unavailable full texts and full-text exclusions. Reporting a more detailed breakdown would require unsupported assumptions.

### 2.3. Data Extraction and Classification

For each publication, the worksheet recorded the year, document type, authors, title, source, Digital Object Identifier (DOI) or record identifier when available, country, author keywords, and abstract. Full texts were used to resolve unclear cases and prepare the qualitative synthesis. In the geographic analysis, each publication received one country category. Because the source export did not preserve the rule used to select among multiple affiliations, the geographic distribution should be read as a description of the worksheet rather than a precise measure of each country’s contribution.

**Figure 2:**
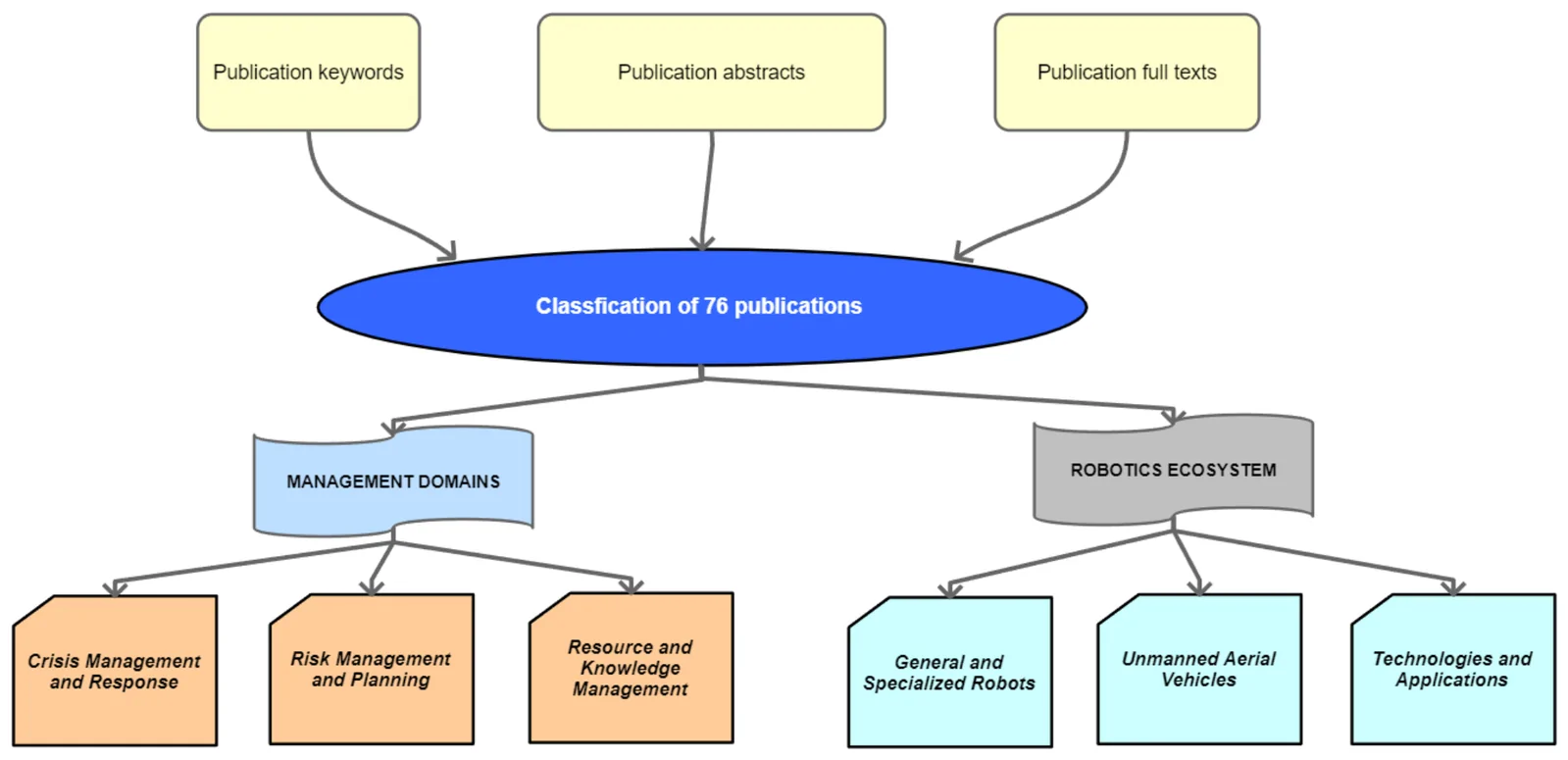
Classification of the publications selected for review according to management domains and the robotics ecosystem.

The two main classification dimensions are shown in Figure 2. Management Domains describe the management purpose of a study. Crisis Management and Response covered the keywords Disaster Management, Crisis Management, Search and Rescue, and Situation Awareness. Risk Management and Planning covered Risk Management, Simulation, and Motion Planning. Resource and Knowledge Management covered Human Resource Management, Information Management, and Environmental Management. These labels were checked against the abstract and, when necessary, the full text rather than assigned from an isolated keyword without context.

The Robotics Ecosystem describes the technical means used in the study. General and Specialized Robots included general, intelligent, industrial, multipurpose, rescue, mobile, agricultural, social, nanorobot, and visually guided robot terms. Unmanned Aerial Vehicles included UAV, drone, unmanned aerial vehicle, and aerial vehicle terms. Technologies and Applications included robot programming and learning, simulation, multi-robot and swarm systems, HRI, rescue operations, robot applications, cameras, sensors, WSNs, and the IoT. A publication could receive more than one ecosystem label when it combined a platform with supporting technologies.

Research methodology was assigned manually from the abstract and article content. Experiment denoted a study that conducted and reported a test or measurement. Literature Analysis denoted a study based primarily on published literature. Case Study covered a developed and implemented solution or an analysis of a specific real case. Conceptual covered a proposed model, method, or system concept, including modelling studies. Survey was used when the evidence came from a questionnaire-based study. Document type was taken from the source database record and grouped as Article, Conference Paper, or Other, with reviews and book chapters placed in Other.

The classifications answer different questions. Management Domains describe the main management purpose served by a study—direct response, risk and planning, or resource and knowledge management—whereas Robotics Ecosystem categories describe the technical means used: physical robots, UAVs, or supporting technologies and applications. The dimensions were coded independently and can intersect. For example, a UAV is a platform category, but its imagery may support immediate response, update a risk map, and later become part of a shared knowledge resource. Table 4 examines these intersections using one dominant management domain per study while retaining multi-label Robotics Ecosystem coding.

Document type and country were mutually exclusive categories. Management Domains, the Robotics Ecosystem, and Research Methodology were coded with multiple labels because one publication could address several functions, technologies, or forms of validation. Their totals may therefore exceed 76. For the qualitative analysis and Table 4, one dominant management domain was additionally assigned according to the publication’s primary purpose. Robotics Ecosystem and methodology labels could still overlap.

Coding used the category definitions reported above and proceeded from the bibliographic record to the full text when the abstract was insufficient. Management labels were assigned from the purpose of the study, Robotics Ecosystem labels from the platform or enabling technology examined, and methodology labels from the evidence actually reported rather than from the terminology used by the authors. A dominant management domain was recorded only for the cross-tabulations and qualitative organization; the multi-label codes were retained separately. Ambiguous assignments were resolved by rereading the relevant passages and checking them against the same operational definition used for the remaining publications.

The coding decision followed a fixed order. First, the study objective and intended crisis-management task were used to identify the management purpose. Second, the physical platform and enabling technologies were coded independently. Third, the reported form of evidence was used to assign the methodology label. A label required support in the title, abstract, methods, results, or full-text description; it was not assigned from a possible future application alone. When a study addressed several management purposes, the dominant domain was the one most closely aligned with its stated objective and principal reported outcome.

The retained worksheet contains the final classifications but not a second, independently coded version of the dataset. A retrospective inter-coder agreement coefficient cannot therefore be calculated from the available records. We do not report an unsupported coefficient or describe the coding as independently validated. Instead, the manuscript provides operational definitions, explains the decision sequence, lists the publications assigned to each recurrent limitation, and includes the reverse publication-by-limitation matrix in Appendix A. The absence of independent double coding remains a methodological limitation and should be addressed prospectively in a future update.

### 2.4. Quantitative Analysis

All calculations were based on 76 unique publications, and percentages used that number as the denominator. Document type and country were mutually exclusive, so a publication could appear in only one row of each of those groups. The remaining dimensions used multiple labels; the same publication could therefore appear in several categories, and their totals and percentages were not required to add to 76 and 100%, respectively.

Seven Pearson chi-square statistics are displayed in Table 1, Table 3 and Table 4. Two comparisons use mutually exclusive publication-level categories: Document Type in Table 1 and Country in Table 3. The other five comparisons involve Management Domains, Robotics Ecosystem labels, or Research Methodology labels, for which one publication may contribute to more than one category. Those label counts are not independent observations. Their chi-square statistics and *p*-values are therefore retained only as exploratory summaries of the cross-tabulated labels and are not used as confirmatory tests of association.

For the two mutually exclusive comparisons, the null hypothesis concerned the association between publication period and the category distribution. Even these results require caution where expected cell counts are small. For the five multi-label comparisons, no claim of formal hypothesis confirmation or equivalence is made, and the *p*-values are not interpreted as proof that relationships are absent. No multiple-testing correction was applied because the analyses are descriptive and exploratory rather than a confirmatory family of tests. The discussion is based primarily on counts, proportions, and the qualitative synthesis.

The Pearson calculations are retained to document the original cross-tabulations, not because the multi-label data satisfy the assumptions of a confirmatory chi-square test. Replacing the five overlapping-label comparisons with several binary regression models would also be unstable in a sample of 76 publications with sparse categories. We therefore do not use the multi-label *p*-values to support substantive conclusions. A larger record-level dataset could use separate binary outcomes or marginal models that account explicitly for repeated labels within publications.

### 2.5. Qualitative Synthesis

The qualitative analysis focused on the task performed by the robot, the capabilities available to it, and the way its operation could support decisions. The review recorded mission conditions, platform characteristics, sensing and communication, the degree of autonomy, HRI, the testing environment, and the maturity of the evidence. Publications were first read within the three dominant management domains and then compared from the Robotics Ecosystem perspective. These were two views of the same 76 studies, not separate publication sets.

Limitations reported by the authors were recorded alongside constraints directly demonstrated by the study design, such as evaluation limited to simulation, a short controlled test, or the absence of a field trial. Coding was binary at publication level: a category was marked only when the paper explicitly described the issue or when the reported design provided direct evidence of it. A limitation was not assigned merely because it is common in robotics. The ten operational categories covered field validation (L1), perception and localization (L2), real-time computation (L3), communication (L4), autonomy and replanning (L5), reliability in harsh conditions (L6), integration and trustworthy information flow (L7), scaling and multi-robot coordination (L8), human–robot collaboration (L9), and energy, hardware, or structural constraints (L10). One publication could receive several codes.

Table 6 reports both the frequency and the complete list of publications assigned to each limitation. Appendix A presents the same coding in the opposite direction, listing the limitation codes assigned to each publication. Together, these two views make every frequency traceable to the included studies. The matrix documents the final coding decisions; it does not replace the missing independent second coding needed for an agreement statistic.

## 3. Results

The final analysis included 76 unique publications issued between 2006 and 2025. Fifteen studies (19.7%) appeared in 2006–2015 and 61 (80.3%) in 2016–2025. The later period therefore contained slightly more than four times as many publications. The results are presented as changes in the publication profile over time, the geographic distribution, and relationships among management domains, the Robotics Ecosystem, and research methodology.

Table 1, Table 3 and Table 4 display seven chi-square statistics. The Document Type and Country comparisons use mutually exclusive categories. The remaining five concern multi-label coding and are shown only as exploratory summaries. Accordingly, the descriptive counts and proportions are the main results, and the multi-label *p*-values are not used to establish or exclude an association.

### 3.1. Publication Profile and Changes Between Periods

Table 1 organizes the publications by document type, management domains, Robotics Ecosystem, and research methodology. Document type is mutually exclusive, whereas the other three groups use multiple labels. A publication may therefore appear in several rows of the same group, and each share is the percentage of all 76 publications assigned that label; the shares are not expected to total 100%.

**Table 1:** Publications by period across all categories.

Name	2006–2015	2016–2025	All Years	Share [%]	Chi-Square
Total	15	61	76	100.0	*χ* ^2^
**Document Type**					
Article	2	14	16	21.1	0.94 (df = 2 *p* = 0.625)
Conference Paper	12	45	57	75	
Other	1	2	3	3.9	
**Management Domains**					
Crisis Management and Response	8	42	50	65.8	0.63 (df = 2 *p* = 0.730)
Risk Management and Planning	3	11	14	18.4	
Resource and Knowledge Management	6	20	26	34.2	
**Robotics Ecosystem**					
General and Specialized Robots	9	38	47	61.8	0.04 (df = 2 *p* = 0.978)
Unmanned Aerial Vehicles	3	14	17	22.4	
Technologies and Applications	9	36	45	59.2	
**Research Methodology**					
Experiment	5	30	35	46.1	2.34 (df = 4 *p* = 0.673)
Literature Analysis	1	8	9	11.8	
Case Study	6	17	23	30.3	
Conceptual	10	42	52	68.4	
Survey	0	3	3	3.9	

**Figure 3:**
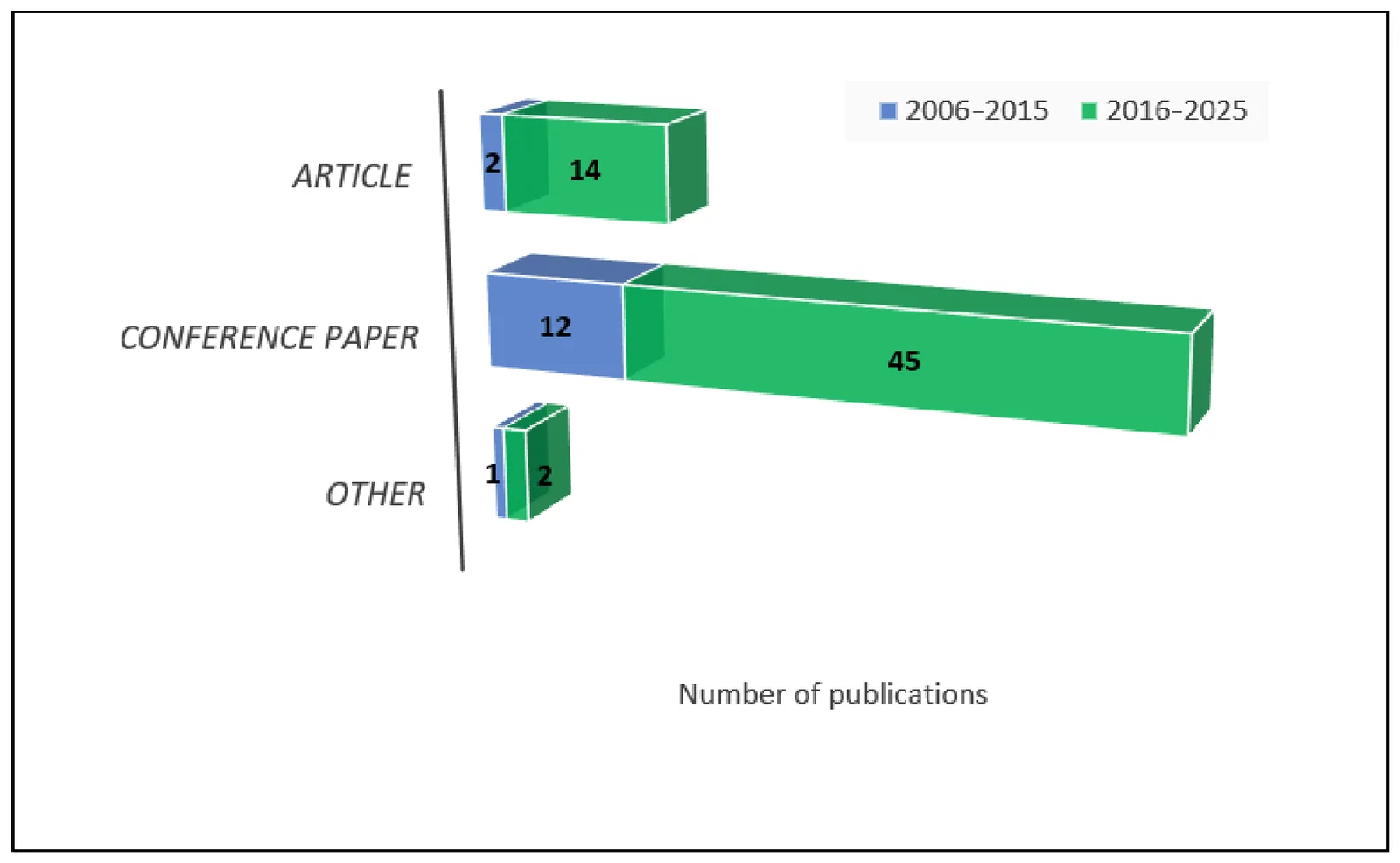
Number of publications by document type in 2006–2015 and 2016–2025.

Conference papers were the dominant document type, accounting for 57 records (75.0%). The reviewed set also contained 16 journal articles (21.1%) and three publications classified as Other (3.9%): two review articles and one book chapter. Between the two periods, the number of conference papers rose from 12 to 45 and the number of journal articles rose from 2 to 14. Despite the increase in volume, the relative distribution of document types did not differ significantly between periods (χ^2^ = 0.94; df = 2; *p* = 0.625), so H_0_ was not rejected. The two-period distribution is illustrated in Figure 3.

Table 2 breaks the overall pattern down by publication year. No reviewed study was published in 2006, 2012, or between January and 20 July 2025. The largest annual counts occurred in 2017 and 2018, with 10 publications in each year, followed by 2019 and 2020 with 9 each. Seven studies appeared in 2021, the count fell to 3 in both 2022 and 2023, and rose to 7 in 2024. The absence of a 2025 record should be interpreted in light of the partial-year cutoff. The annual totals reproduce the period counts of 15 publications in 2006–2015 and 61 in 2016–2025.

**Table 2:** Number of reviewed publications by year.

Year	Publications	Year	Publications
2006	0	2016	3
2007	2	2017	10
2008	1	2018	10
2009	1	2019	9
2010	3	2020	9
2011	2	2021	7
2012	0	2022	3
2013	1	2023	3
2014	2	2024	7
2015	3	2025	0

**Figure 4:**
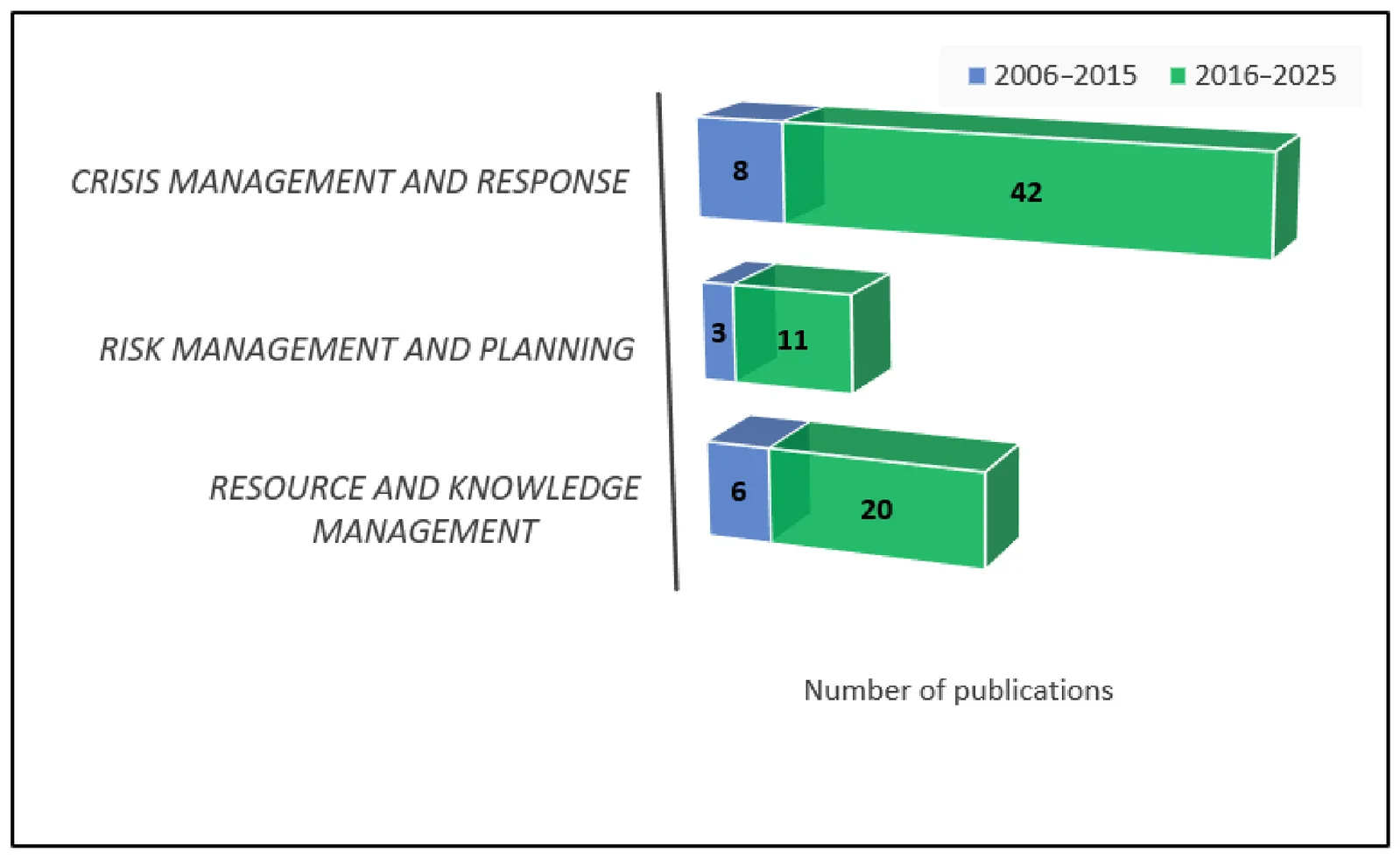
Number of publications by management domains in 2006–2015 and 2016–2025.

Crisis Management and Response was the most frequent management label, appearing in 50 publications (65.8%). Resource and Knowledge Management was identified in 26 studies (34.2%), and Risk Management and Planning in 14 (18.4%). After 2015, the counts increased in all three areas: from 8 to 42, from 6 to 20, and from 3 to 11 publications, respectively. The exploratory multi-label comparison produced χ^2^ = 0.63, df = 2, and *p* = 0.730. Because publications could contribute to more than one management label, this value is reported descriptively and is not treated as evidence that the distributions are equivalent. Figure 4 presents the corresponding counts.

**Figure 5:**
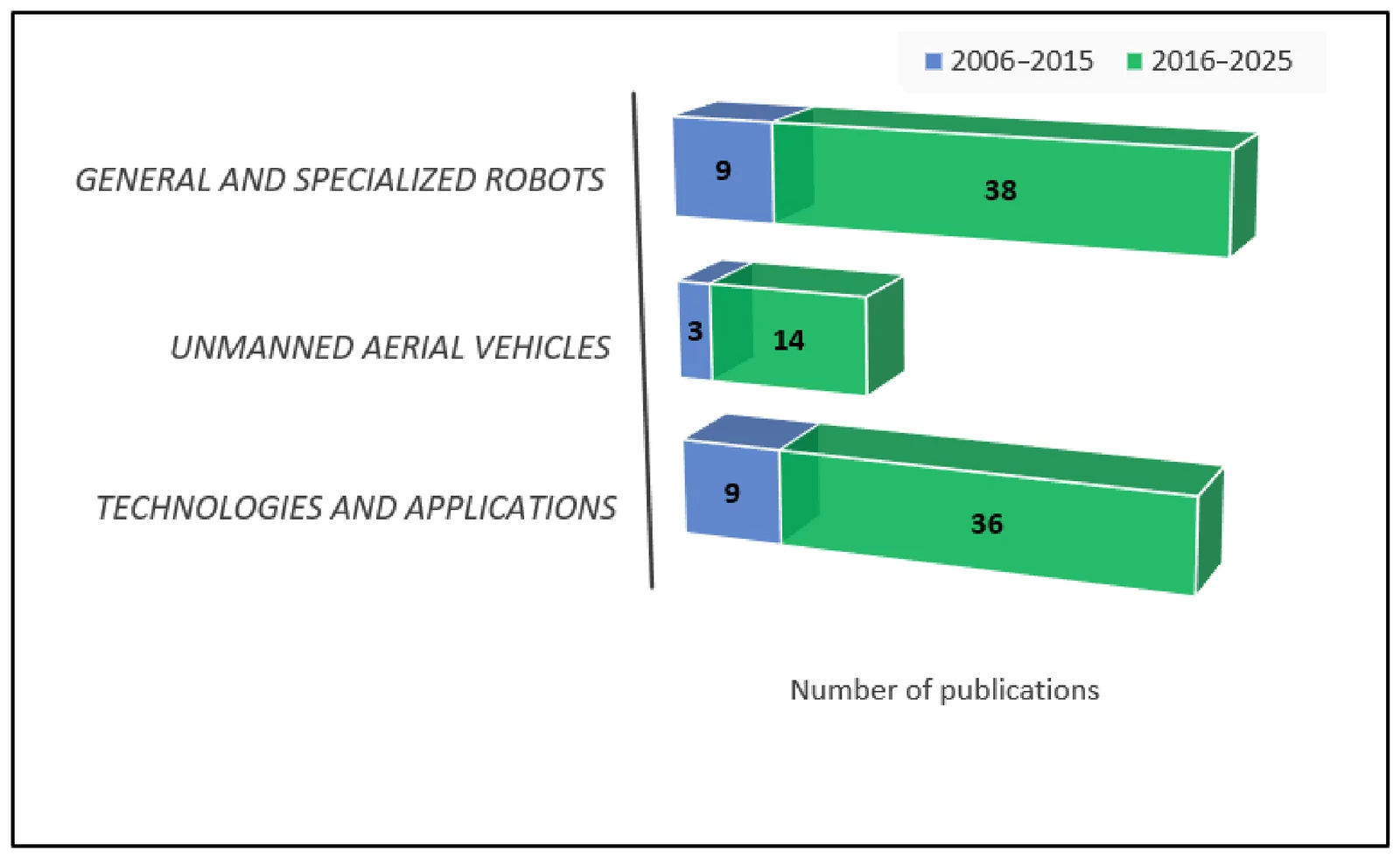
Number of publications in Robotics Ecosystem categories in 2006–2015 and 2016–2025.

Within the Robotics Ecosystem, General and Specialized Robots appeared in 47 publications (61.8%), Technologies and Applications in 45 (59.2%), and UAVs in 17 (22.4%). The period counts were 9 and 38, 9 and 36, and 3 and 14, respectively. The exploratory multi-label comparison produced χ^2^ = 0.04, df = 2, and *p* = 0.978. This *p*-value does not establish stability or equivalence because individual publications could receive several ecosystem labels. The descriptive distributions are shown in Figure 5.

**Figure 6:**
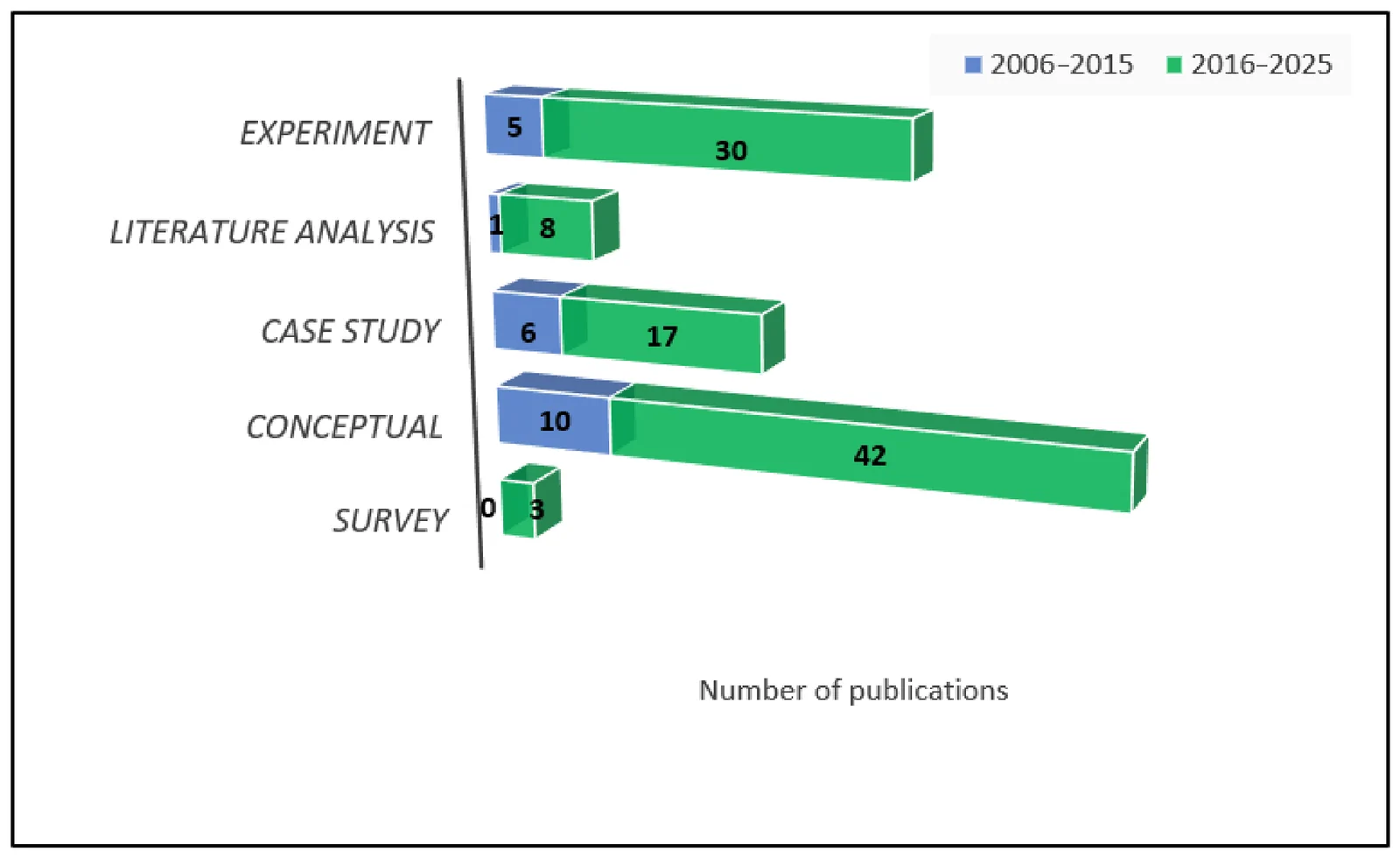
Number of publications by research methodology in 2006–2015 and 2016–2025.

Conceptual research was the most frequent methodological category, appearing in 52 publications (68.4%). Experiments were identified in 35 studies (46.1%), case studies in 23 (30.3%), literature analyses in 9 (11.8%), and surveys in 3 (3.9%). Every category contained more publications in the later period, although surveys appeared only after 2015. The exploratory multi-label comparison produced χ^2^ = 2.34, df = 4, and *p* = 0.673. Since the same publication could receive several methodology labels, the counts and proportions, rather than this *p*-value, provide the basis for interpretation. Figure 6 summarizes the methodological counts.

### 3.2. Geographic Distribution of Publications

**Figure 7:**
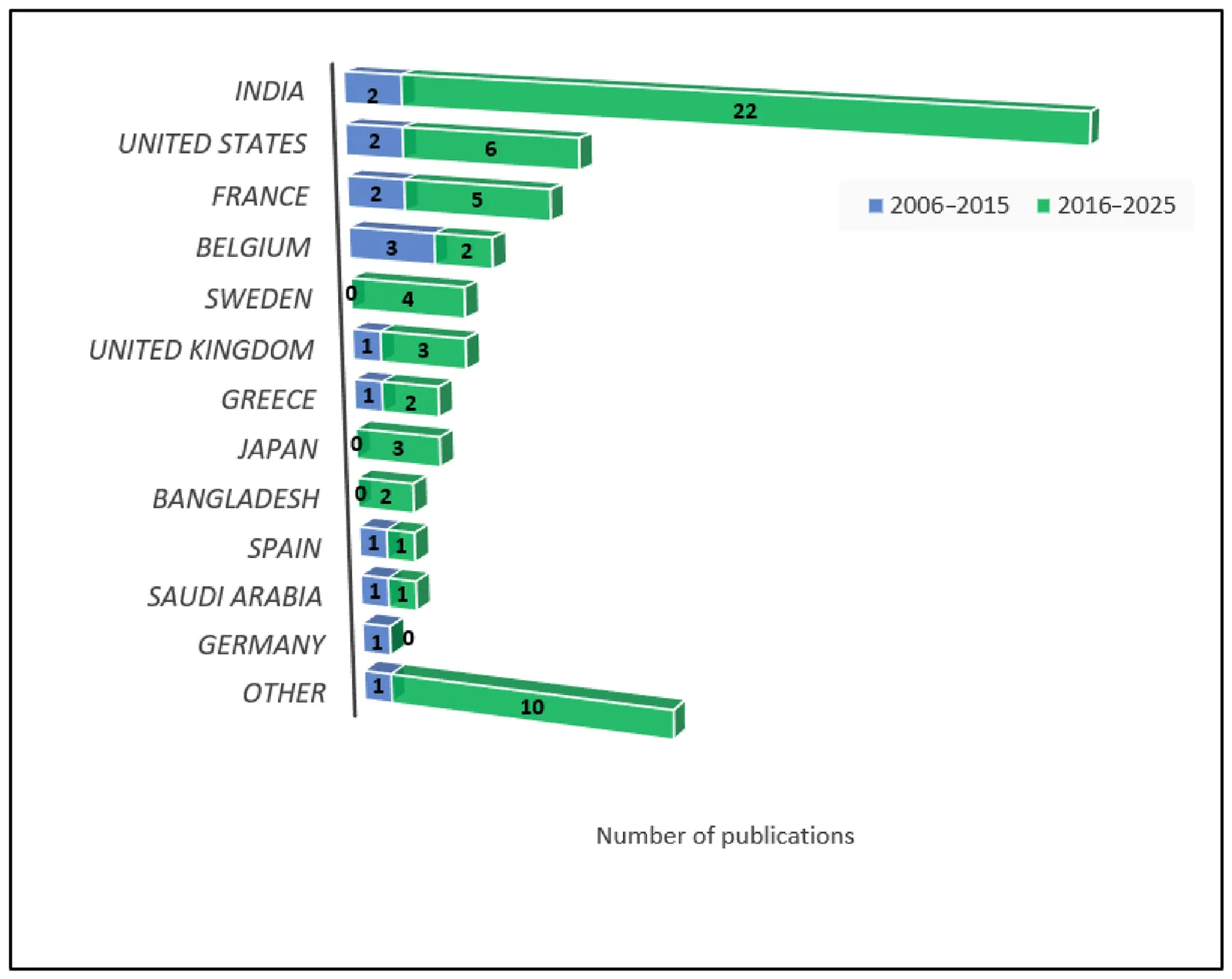
Number of publications by affiliation country in 2006–2015 and 2016–2025.

For the geographic analysis, each publication was assigned one country category from the affiliation recorded in the worksheet. India contributed the largest number, with 24 publications (31.6%). It was followed by the United States with 8 (10.5%), France with 7 (9.2%), and Belgium with 5 (6.6%). The Other category included 11 publications (14.5%) from countries represented by one or only a few records. The country distribution is shown in Figure 7.

The largest numerical change occurred in India, where the number of publications increased from 2 in 2006–2015 to 22 in 2016–2025. The count rose from 2 to 6 in the United States and from 2 to 5 in France. Sweden, Japan, and Bangladesh were represented only in the later period, while Germany appeared only in the earlier one. Detailed counts and shares are provided in Table 3.

**Table 3:** Publications by period and affiliation country.

Country	2006–2015	2016–2025	All Years	Share [%]	Chi-Square
All countries	15	61	76	100.0	***χ^2^***
India	2	22	24	31.6	17.37 (df = 12, *p* = 0.136)
United States	2	6	8	10.5	
France	2	5	7	9.2	
Belgium	3	2	5	6.6	
Sweden	0	4	4	5.3	
United Kingdom	1	3	4	5.3	
Japan	0	3	3	3.9	
Greece	1	2	3	3.9	
Bangladesh	0	2	2	2.6	
Spain	1	1	2	2.6	
Saudi Arabia	1	1	2	2.6	
Germany	1	0	1	1.3	
Other	1	10	11	14.5	

The comparison of country and period produced χ^2^ = 17.37; df = 12; *p* = 0.136. Because *p* was greater than 0.05, the observed geographic differences were not statistically significant. They should be treated as a description of the publications included in the review, not as evidence of a broader geographic shift in the field.

### 3.3. Relationships Among Management Domains, the Robotics Ecosystem, and Research Methodology

Table 4 presents the same 76 publications from another perspective. Each study was assigned one dominant management domain: 30 publications to Crisis Management and Response, 21 to Risk Management and Planning, and 25 to Resource and Knowledge Management. The Robotics Ecosystem and Research Methodology continued to use multiple labels. Totals in those parts of the table may therefore exceed both 76 and the number assigned to the dominant domain in a given column.

**Table 4:** Distribution of Robotics Ecosystem and Research Methodology labels across dominant management domains.

Name	Crisis Management and Response	Risk Management and Planning	Resource and Knowledge Management	Total	Chi-Square
**Total**	**30**	**21**	**25**	**76**	**χ^2^**
**Robotics Ecosystem**					
**General and Specialized Robots**	21	8	18	47	1.72 (df = 4, *p* = 0.787)
**Unmanned Aerial Vehicles**	6	5	6	17	
**Technologies and Applications**	16	11	18	45	
**Research Methodology**					
**Experiment**	15	10	10	35	2.82 (df = 8, *p* = 0.945)
**Literature Analysis**	3	3	3	9	
**Case Study**	9	7	7	23	
**Conceptual**	20	12	20	52	
**Survey**	2	0	1	3	

Conceptual research was the most frequent methodological approach in every management domain: 20 publications in Crisis Management and Response, 12 in Risk Management and Planning, and 20 in Resource and Knowledge Management. Experiments appeared in 15, 10, and 10 publications, respectively, and case studies in 9, 7, and 7. Literature reviews were evenly distributed, with three publications in each domain. Surveys were used in only three studies: two in Crisis Management and Response and one in Resource and Knowledge Management.

**Figure 8:**
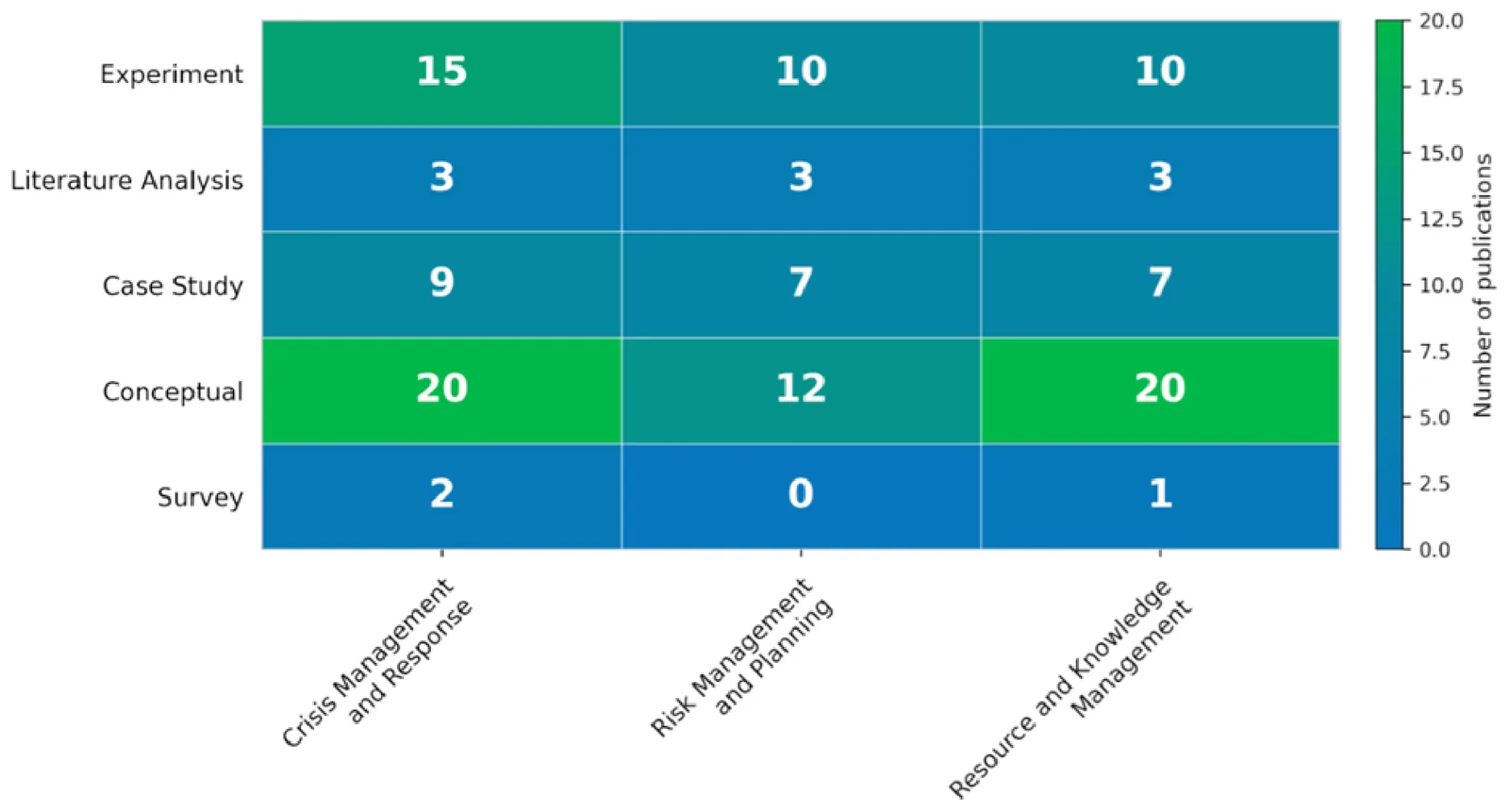
Counts of Research Methodology labels by dominant management domain. Labels may overlap.

Figure 8 displays the label counts without treating the colour differences as evidence of association. Conceptual studies were most frequent in the first and third domains. The reported value (χ^2^ = 2.82, df = 8, *p* = 0.945) is an exploratory summary because publications could receive several methodology labels.

General and Specialized Robots were identified in 21 of the 30 Crisis Management and Response publications, 8 of the 21 Risk Management and Planning publications, and 18 of the 25 Resource and Knowledge Management studies. UAVs were distributed more evenly across the three domains, appearing in 6, 5, and 6 publications, respectively. Technologies and Applications appeared in 16, 11, and 18 publications. The row totals—47, 17, and 45—are the same as those in Table 1.

**Figure 9:**
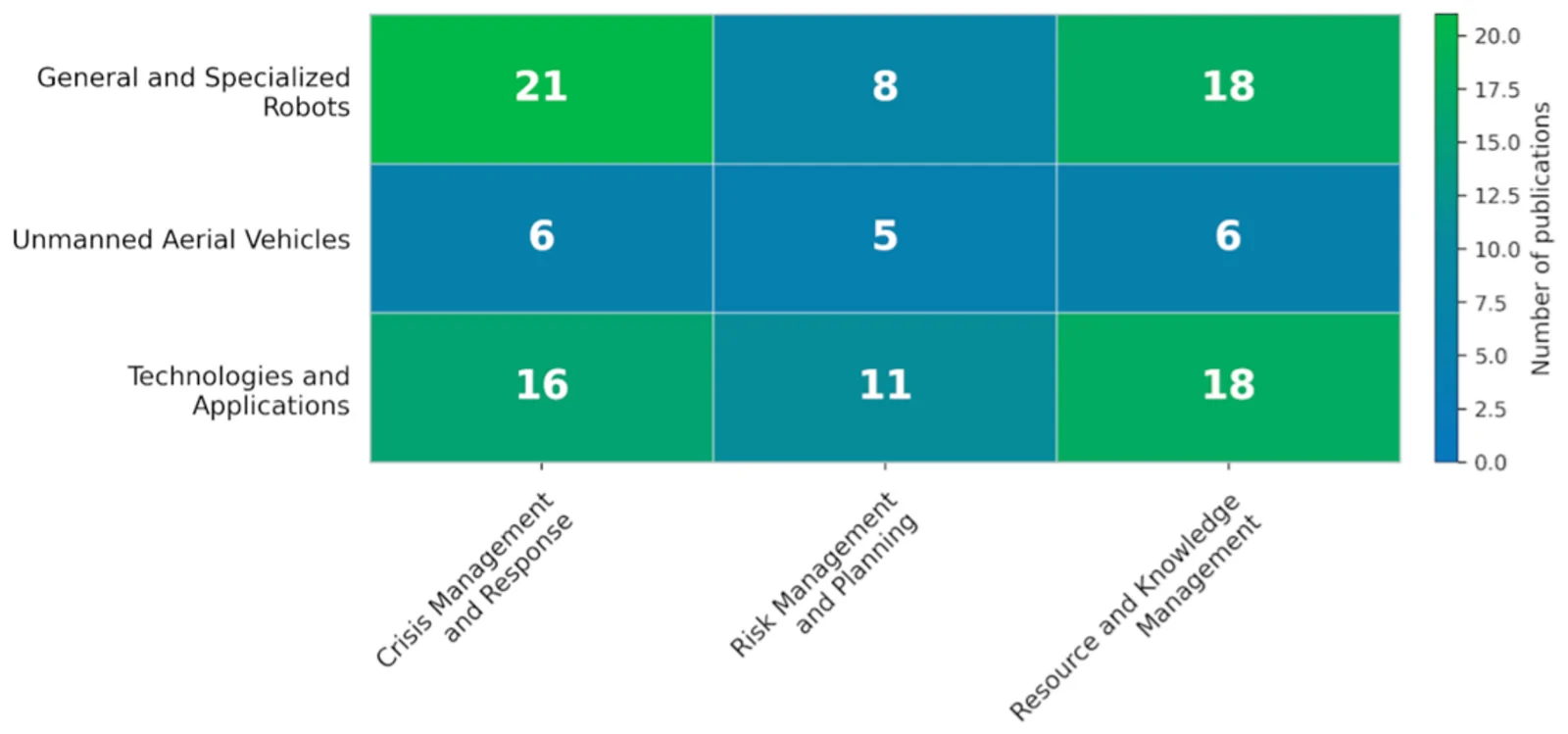
Counts of Robotics Ecosystem labels by dominant management domain. Labels may overlap.

Figure 9 displays the ecosystem label counts. General and Specialized Robots and Technologies and Applications appeared more often than UAVs in each domain. The reported value (χ^2^ = 1.72, df = 4, *p* = 0.787) is exploratory and does not establish independence between technology and management purpose.

Publication volume increased markedly after 2015, while conference papers remained the main format. The categories examined most often were Crisis Management and Response, General and Specialized Robots, Technologies and Applications, and conceptual research. India was the most frequently represented affiliation country.

None of the seven reported chi-square statistics had a *p*-value below 0.05. This observation is not treated as proof of equivalence or as evidence that no relationships exist. Two comparisons concern mutually exclusive categories, while five are exploratory summaries of overlapping labels. The descriptive distributions and qualitative synthesis therefore remain the main basis for the discussion.

## 4. State of the Art

Section 3 showed how the literature changed over time, which categories occurred most often, and whether their distributions differed between publication periods or Management Domains. Section 4 turns from counts to content and addresses RQ4: what the systems were designed to do, how they were evaluated, and when a technical capability can provide practical support during a crisis.

For this part of the review, each publication was placed in the Management Domain that best matched its main purpose: 30 in Crisis Management and Response, 21 in Risk Management and Planning, and 25 in Resource and Knowledge Management. The categories organize the discussion without separating activities that remain connected during a real mission. Reconnaissance may change a plan, the plan influences how resources are used, and communication determines whether observations become shared knowledge.

Section 4.4 returns to the same publications from the cross-cutting perspective of the Robotics Ecosystem. Overlap is expected here because one system may combine a mobile platform, a UAV, sensors, path-planning software, communication, and HRI. The dominant Management Domain counts therefore sum to 76, whereas the multi-label totals reported in Table 1 and Table 4 may exceed that number.

### 4.1. Crisis Management and Response

During direct response, a robot mainly extends the physical and informational reach of emergency services. It may approach a source of danger, search a confined space, measure contamination, or provide a view of an area before people enter it. The literature does not support replacing command authority or human responsibility with autonomous machines. It supports a more limited and practical role: reducing exposure, shortening reconnaissance time, and collecting data that can guide the next decision [34,35].

The 30 publications assigned to this domain cover a broad technical range, from inexpensive remotely controlled prototypes to multi-platform systems that combine aerial and ground vehicles. Read together, they show that response robotics has moved beyond the idea of a single machine sent into a hazardous area. The more mature systems link mobility, sensing, mapping, communication, and operator support. Their value therefore depends on the quality of the entire response chain, not only on whether the robot can move.

Several designs begin with a simple operational question: can a robot establish whether an area contains a threat or a person who needs help? The Disaster Response and Surveillance Bot (DRASB) combines wireless control with gas, flame, temperature, and motion sensing [36]. Other low-cost prototypes use passive infrared, ultrasonic, temperature, or gas sensors to identify human presence and environmental hazards. Some also add a camera, smartphone control, a manipulator, modular payloads, or the ability to travel between land and water [37,38,39,40]. These solutions are attractive because they can be built with widely available components, but most were demonstrated in simplified environments and over short communication distances.

A more specialized platform is designed around the constraints of the environment it must enter. The Advanced Robot for Extraction and Survey (ARES) combines a tracked or wheeled base, video, and image segmentation to support local assessment [41]. Stereo vision has also been used to combine victim detection with terrain-traversability analysis so that perception directly influences navigation [42]. These studies make an important distinction between seeing an object and deciding whether the robot can safely reach it. In debris, a useful detector must work together with mobility and route assessment.

Hazard sensing creates a similar integration problem. A mobile gas-sensing robot combined a gas-sensor module, human detection, obstacle sensing, and Global Positioning System (GPS) coordinates so that measurements could be associated with locations on the terrain [10]. The reported classifier distinguished selected gases during tests on uneven ground, but its operational value would still depend on calibration, sensor drift, cross-sensitivity, and communication in a contaminated environment. A measurement becomes actionable only when the response team knows where it was taken, when it was taken, and how reliable it is.

Confined spaces require a different mechanical solution. A continuum robot equipped with 80 distributed sensors was developed to enter narrow voids while collecting inertial, acoustic, vibration, and communication data [11]. The work is notable because it treats sensing and mechanical flexibility as one design problem. Adding more sensors can improve search efficiency, but wiring, noise, maintenance, and stiffness may reduce the very mobility that makes a continuum platform useful. This trade-off is especially important in collapsed structures, where the robot must remain maneuverable after repeated contact with debris.

UAVs address the opposite scale. Instead of entering a narrow void, they can cover a large or inaccessible area quickly. Sensor fusion between infrared imaging and LiDAR has been used to support victim search in environments without GPS [13]. A wildfire-assistance concept combined aerial observation with an interactive dashboard showing the estimated location of people and the spread of a fire [14]. A landslide-response system proposed a heterogeneous group of flying, crawling, and penetrating robots to combine wide-area observation with close inspection [15]. These examples show why aerial and ground platforms are complementary: the UAV identifies where attention is needed, while another robot or a human team examines the location in greater detail.

Raw sensor data is rarely sufficient for a response commander. Video, point clouds, thermal images, and chemical readings must be converted into a form that helps distinguish a safe route, a likely victim location, or a structural hazard. The VIEW-FINDER project linked robots carrying chemical sensors, cameras, and laser scanners with a base station and a crisis-management information system [43]. Its significance lies less in any individual sensor than in the attempt to combine on-site data with geographic information and deliver it to the people directing the operation.

Image quality is a practical constraint in moving platforms. Motion blur, poor lighting, smoke, and unstable viewpoints can degrade detection before an algorithm begins to interpret the scene. Work on image deblurring and localization used enhancement and detection methods to identify people from moving-camera footage without relying on an Internet connection [44]. The study illustrates an operational advantage of local processing, but it also shows how several errors can accumulate: poor input affects enhancement, enhancement affects detection, and detection affects the estimated location.

Three-dimensional scene understanding has been approached through both distributed learning and incremental mapping. A view-invariant convolutional neural network (CNN) was proposed to recognize objects from depth information in disordered environments without depending on RGB imagery [45]. Incremental segmentation of point clouds from disaster sites aimed to build a semantic 3D map as laser scans arrived, rather than waiting for post-processing after the mission [46]. In both cases, the central issue is timeliness. A highly accurate map that reaches the command team too late may be less useful than a coarser result delivered while routes and priorities can still be changed.

The same requirement applies to aerial perception. Victim search with infrared and LiDAR data depends on the alignment of sensors, the motion of the platform, and the ability to navigate where satellite positioning is unavailable [13]. Wildfire observation adds a moving hazard whose boundary may change between successive measurements [14]. Perception should therefore be reported with uncertainty and time information. A map or detection should not appear more certain or more current than the data from which it was produced.

An immersive interface can improve how an operator uses a map, but it also adds technical dependencies. A virtual-reality (VR) system combined red–green–blue plus depth (RGB-D) imaging, three-dimensional reconstruction, simultaneous localization and mapping (SLAM), and remote exploration [21]. The operator could view already reconstructed parts of the environment independently of the robot’s current camera direction. This reduces the tunnel vision of conventional video teleoperation, yet the approach requires stable data transmission, accurate registration, and an interface that remains understandable under time pressure.

Response robotics is still largely a human–machine activity. The operator decides where to look, how much risk to accept, and when an automatic behavior should be interrupted. Research on emergency response in hazardous facilities argues that higher-level robot behaviors, improved sensor-data abstractions, and more suitable control metaphors can reduce workload and exposure [26]. The important point is not to maximize autonomy in every task, but to assign work in a way that preserves human understanding of the situation.

Interface studies show that a technically functional control method may still be unsuitable for crisis work. A tangible tabletop linked physical mini-robots with robots operating in a simulated intervention area [27]. Tangible interaction improved effectiveness, efficiency, and usability compared with touch interaction for the tested tasks, but it did not significantly reduce workload. This distinction matters: an interface can make commands easier to issue without reducing the cognitive effort required to monitor several robots and interpret their data.

Route memory provides a different form of support. A rescue platform that generated a map of its traveled area could return toward its starting point after losing the communication link [47]. This is a modest autonomy feature, but it addresses a realistic failure mode: a robot should not be lost simply because the operator can no longer reach it. Safe fallback behavior, route logging, and a clear indication of what the robot will do after disconnection may be more useful in practice than a complex autonomous function that is difficult to predict.

Gesture-based control has also been explored. A controller integrating conventional analog inputs with Leap Motion sensing was proposed for a search-and-rescue robot [48]. Such interfaces may allow fast and intuitive commands, but they must be tested under the conditions in which responders work: protective clothing, restricted movement, noise, poor visibility, and stress. The quality of control transfer and the possibility of confirming or cancelling a command are at least as important as recognition accuracy in a laboratory.

A single platform is limited by its payload, viewpoint, endurance, and communication range. Multi-robot systems distribute these limitations across several units, but they replace one difficult problem with several coordination problems. Situation Responsive Networking of Mobile Robots for Disaster Management (SENEKA) treated mobile robots and networking as parts of the same response capability [12]. The AWARE architecture combined UAVs, ground cameras, and wireless sensors and demonstrated the transport and deployment of sensor payloads by autonomous aircraft [20]. Both approaches move toward response infrastructure rather than an isolated robot.

Task allocation is especially visible in urban search and rescue. A distributed approach used several robots to clear blocked roads so that fire, ambulance, and other agents could reach affected locations [49]. The work was evaluated in simulation, but the management problem is realistic: clearing one road may improve access for several teams, while choosing the wrong obstacle may consume time without changing the overall response. The value of a robot task should therefore be measured by its effect on the mission, not only by the completion of the local action.

Swarm concepts extend this logic to larger groups of relatively simple units. Proposed systems include coordinated victim search and extraction [50], behavior-based search guided by a virtual leader and fuzzy control [51], and a cooperative model for mine emergencies tested with two to four robots [52]. The attraction of a swarm lies in coverage and redundancy. However, simulated scalability does not guarantee operational scalability, because radio traffic, localization errors, collision avoidance, and inconsistent world models become more difficult as the team grows.

A multi-UAV system for coordinated human detection combined region partitioning with a deep-learning detector in a simulated disaster environment [53]. Separating the search area can reduce duplication and shorten coverage time, but the benefit depends on reliable localization and task handover. A real mission must also cope with a vehicle leaving the team, a detector producing a false alarm, or a newly reported priority requiring the search pattern to be changed.

Across the 30 publications assigned to Crisis Management and Response, the strongest trend is a shift from remotely controlled sensing platforms toward systems that interpret data and cooperate with other devices. The evidence is less consistent when the question changes from technical feasibility to operational readiness. Many prototypes demonstrate one function successfully, while fewer studies test the full chain of deployment, sensing, communication, interpretation, decision, and recovery under deliberately degraded conditions [10,11,12,13,14,15,20,21,26,27,34,35,36,37,38,39,40,41,42,43,44,45,46,47,48,49,50,51,52,53].

### 4.2. Risk Management and Planning

Risk management begins before a robot enters the affected area. It includes recognizing hazards, selecting equipment, preparing routes, allocating tasks, and checking whether the proposed hardware and software can cope with the expected conditions. The 21 publications assigned to this domain show that planning is not a single algorithm. It is a continuing comparison between mission objectives, uncertain environmental information, platform limits, communication, and available energy.

Simulation allows a team to examine failure without risking personnel or equipment. At the organizational level, it can be used to rehearse teamwork, communication, surge capacity, and coordination among institutions. Evidence that simulation improves task performance is stronger than evidence that it improves outcomes during actual disasters, and the link between an exercise and later operational performance remains difficult to establish [24]. This is why simulation should be treated as preparation and diagnosis, not as proof of readiness.

Robot-centered simulation addresses a related but narrower question: is the platform configured for the intended mission? Three-dimensional environments have been used to model the robot, its sensors, geometry, weather, and mission sequence before deployment [55]. A companion study emphasized that errors in sensor configuration can produce inaccurate data and, in turn, poor decisions [56]. These studies make a useful contribution by connecting simulation parameters with mission requirements instead of evaluating an algorithm in an abstract environment.

A virtual playground for smart cyber–physical systems provides another level of testing. It allows developers to deploy multiple simulated devices, obstacles, and environmental elements and observe how software-controlled ensembles behave [57]. This is valuable for systems whose components are expensive or difficult to assemble for every test. At the same time, the simulation remains only as credible as its models of communication loss, sensor noise, collision, terrain, and weather.

A practical validation sequence should therefore move through several stages: software-only simulation, hardware-in-the-loop testing, controlled physical trials, exercises with responders, and field evaluation. Each stage answers a different question. A simulation may reveal a flawed configuration, while a field trial reveals whether connectors fail in dust, a radio link disappears behind concrete, or an operator misunderstands an automated action. Skipping the intermediate stages makes it difficult to determine why a system failed.

Route planning cannot be separated from the mechanical abilities of the platform. A semi-automated hexapod was designed to negotiate unmapped terrain while using cameras, distance sensing, and object recognition [23]. Legged robots can step over obstacles that stop a small wheeled base, but they require stable gait generation, balance, and more complex control. A review of biped stability similarly distinguishes between generating a stable gait and recovering after a disturbance [64]. These are not only mechanical concerns; a fall can interrupt communication, damage sensors, or block a route needed by the mission.

Soft and modular designs address other forms of uncertainty. A fully 3D-printed hydrogel actuator demonstrated contraction behavior suitable for a jellyfish-like soft robot [65]. The study is enabling component research rather than a disaster deployment, but it shows how compliant materials may support movement in water or contact-rich environments. Modular prototypes such as SQ-BOT and 3DoBot aim to reconfigure their structure for different tasks [66,67,68,69]. Their flexibility is promising, although docking reliability, power transfer, control after reconfiguration, and resistance to impact remain open engineering issues.

Snake-like robots offer a narrow profile and many degrees of freedom. One prototype reproduced a slithering motion and accepted voice-derived commands through an infrared link [68]. Another study examined the vibration and impact behavior of additively manufactured serpenoid segments intended for rescue use [67]. Together, these works show why structural testing belongs in risk management. A robot that can theoretically reach a void is not useful if its segments deform, its joints jam, or the communication method fails behind debris.

Transport-oriented studies broaden the planning problem beyond reconnaissance. One publication considered autonomous transport at system level [62]. Another implemented a low-cost GPS-guided carrier that used a digital compass, obstacle sensing, and an automatic return function to deliver small objects to a prescribed destination [63]. In crisis operations, delivery can reduce responder exposure, but GPS availability, payload, terrain, and route security must be evaluated before a laboratory carrier can be treated as a logistical resource.

Platform selection should therefore start from the environment and task rather than from a preferred robot type. A UAV may be the right tool for a rapid overview but not for manipulation. A continuum robot can enter a void but cannot carry the payload of a tracked vehicle. A modular system may adapt its shape, yet spend time and energy on reconfiguration. Risk management requires these trade-offs to be stated before deployment so that capability gaps are not discovered during the mission.

Robots can contribute to risk reduction before an event develops into a full emergency. A system combining an IoT-equipped master quadcopter with subordinate platforms was proposed for monitoring air and water pollution [58]. The concept illustrates how robotic mobility can place sensors where fixed monitoring is absent or insufficient. Its usefulness depends on calibration, sampling strategy, and the ability to compare measurements over time rather than on the flight platform alone.

UAV mapping has also been applied to landslide assessment. A case study in the Dominican Republic used aerial data to examine terrain change, support two- and three-dimensional geographic records, and reduce the need for repeated physical inspection [59]. This is an example of robotics supporting preparedness and urban planning as well as immediate response. Aerial imagery becomes more valuable when it can be compared with earlier surveys and linked to infrastructure, buildings, and known vulnerabilities in a Geographic Information System (GIS).

Wildfire planning requires the route to change as the hazard changes. A hybrid method combining deep reinforcement learning with particle swarm optimization was evaluated in simulated forest-fire scenarios [60]. The reported results indicate the potential for rapid adaptation, but simulated success rates do not establish performance in smoke, wind, heat, and unreliable communication. A planning method should be evaluated not only on travel time, but also on safety margins and the consequences of delayed or incorrect fire-front information.

When the environment is initially unknown, a robot must decide where the next measurement is most valuable. An informative-path-planning algorithm estimated the expected mapping gain of different subareas and used that estimate to guide local navigation [61]. Its performance was compared with a benchmark that assumed prior knowledge of the environment. This distinction between an implementable heuristic and an ideal benchmark is useful because it shows how much efficiency is lost when the robot must work with partial information.

In a multi-robot mission, the shortest individual paths may not produce the best collective outcome. A potential-field method combined with simulated annealing was proposed to avoid obstacles and reduce the local-minimum problem during simultaneous planning for several robots [54]. The method also included multi-task allocation. Such approaches are useful in dynamic environments, but their performance depends on obstacle detection, map updates, and the assumptions used to represent the movement of other robots.

Multi-objective allocation makes the underlying trade-offs explicit. The Analytic Hierarchy Process (AHP) was used to weight total distance, the longest route, and workload balance before comparing market-based, Robot and Task Mean Allocation (RTMA), and balanced assignment approaches [70]. The results show that the preferred solution changes with the objectives and the size of the simulated problem. In practice, the weights should reflect mission priorities and may need to change when a new hazard or victim location is reported.

Formation planning addresses the geometry of the team. A distributed mechanism allowed unmanned ground vehicles (UGVs) to select, scale, and transform formations when the original arrangement could not be maintained around obstacles [71]. Communication planning adds another constraint. A constraint-based local-search approach coordinated mobile sensing robots and relay units so that data could continue to reach an operating center despite uncertainty in travel time [72]. These studies show that movement and connectivity should be planned together.

Across the 21 publications in Risk Management and Planning, a pre-mission plan is best understood as an initial hypothesis. It must be revised as new information arrives, equipment condition changes, and communication deteriorates. The next research step is not only to produce a shorter route or a better simulated allocation, but to show how the plan degrades safely, how priorities are updated, and how the operator can understand why the system changed its decision [23,24,54,55,56,57,58,59,60,61,62,63,64,65,66,67,68,69,70,71,72].

### 4.3. Resource and Knowledge Management

In a crisis operation, resources include robots, sensors, batteries, radio channels, processing capacity, and operator attention. Information is also a resource. It has operational value only when its origin is known, its age is visible, and it reaches the right participant before conditions change. The 25 publications assigned to Resource and Knowledge Management examine how data is transmitted, combined, described, protected, and presented to people and machines.

Fixed communication infrastructure may be damaged or unreachable during a disaster. A multi-agent WSN was proposed to monitor an incident, track responders and robots, aggregate data, and manage battery use without depending on a cellular base station [16]. A First Responders Robotic Network treated robots as temporary network nodes that could cover intervention areas, connect physical paths, and support several search-and-rescue teams [17]. These approaches make communication itself a deployable resource.

Heterogeneous sensors and robots also require gateways that can be configured during the event. A distributed Internet of Things (IoT) gateway architecture used network functions virtualization (NFV) and software-defined networking (SDN) to provision gateway functions and connect devices dynamically [18]. The prototype demonstrates architectural feasibility, but operational use would require rapid configuration, clear responsibility for network control, and graceful behavior when some nodes disappear.

Security cannot be added after the network has been deployed. Work on multi-robot communication identifies threats and security functions across the network stack, including communication between robots and between robots and infrastructure [19]. Authentication, confidentiality, and integrity are particularly difficult in a changing team because devices join, leave, and reconnect. A system that rejects a legitimate replacement unit or accepts a compromised node can disrupt the mission even if the radio link itself remains available.

Wide-area connectivity offers another option. Cellular IoT has been considered as a way to connect robots, UAVs, and wireless sensors during large-scale disasters and to support recovery when local infrastructure is partially available [73]. It can extend range, but it does not remove dependence on external infrastructure or service availability. A resilient design should be able to switch between local, ad hoc, relay-based, and wide-area communication rather than treating one technology as universally available.

Transmitting observations is not the same as building shared knowledge. Robots may use different coordinate frames, object labels, update rates, and levels of detail. A distributed semantic framework was developed so that heterogeneous robots could share task-relevant knowledge without relying on a central cloud service [30]. The comparison with a centralized approach is important because centralization simplifies consistency, while distribution reduces latency and dependence on a single node.

Hastily Formed Knowledge Networks (HFKNs) extend this idea to teams of robots and people. The framework described by Doherty et al. connects low-level sensor data with higher-level semantic knowledge represented in Resource Description Framework (RDF) graphs [29]. It supports distributed queries and was demonstrated in a field mapping case involving several UAVs. The study shows how knowledge management can become part of a mission rather than a database operation performed afterward.

The RGS⊕ system focuses more directly on synchronizing RDF graphs among agents that may communicate intermittently [28,32]. Local graphs remain available while knowledge is collected, and synchronization resumes when connections permit. This is valuable in emergencies because continuous access to a central store cannot be assumed. It also creates a difficult reconciliation problem: after disconnection, two agents may hold different descriptions of the same object, location, or event.

Cooperative tracking requires a related form of reconciliation at the estimation level. A delayed-state information filter combined data from heterogeneous static and mobile sensors while accounting for communication delay and retaining information about earlier states [31]. The approach reduces dependence on a central estimator, but maintaining trajectories and exchanging delayed information increases message size and synchronization demands. This is a clear example of the trade-off between estimation quality and communication cost.

Trust and provenance become more difficult as the team grows. A blockchain-based perspective on robotic swarms discusses how consensus and tamper-resistant records could support accountability and information sharing [33]. The approach is conceptual and introduces its own burdens: computation, storage, latency, and energy. In crisis robotics, a trust mechanism is useful only if it can operate within the limits of the platforms and does not delay time-critical decisions.

Environmental monitoring illustrates how sensing, mobility, and data management meet. A quadruped robot combined air-quality sensors, positioning, a robot operating framework, and a database to collect and transmit measurements in an urban environment [74]. Pipeline monitoring research similarly compares robot and WSN configurations, sensor coverage, communication methods, and maintenance requirements [75]. These systems are relevant to crisis management because they can identify deterioration or leakage before it develops into a larger incident.

Remote sensing can extend the scale of measurement. Hyperspectral cameras and LiDAR-based methods have been evaluated for detecting, visualizing, and quantifying gas leaks [82]. Such equipment may be carried by a vehicle or used from a stand-off position. The management challenge is to connect a plume estimate with location, wind, uncertainty, and response thresholds so that technical measurements can support evacuation, isolation, or inspection decisions.

A reconfigurable smart sensor interface can reduce the effort needed to connect different sensors to a robot. One study used a field-programmable gate array and an IEEE 1451.2-based interface to acquire data in an IoT environment [84]. Multi-UAV mapping offers a different form of resource sharing: several small aerial vehicles with simple cameras were organized in formation to reconstruct a three-dimensional scene that would normally require a heavier sensor [85]. Both approaches seek capability through integration rather than through one more powerful device.

Cloud-based Robots-as-a-Service provides processing and orchestration outside the platform [77]. It can offer unified descriptions, discovery, and federation of heterogeneous robots during a large incident. The advantage is access to resources beyond the robot’s onboard computer. The disadvantage is dependence on communication, remote infrastructure, and service management. A cloud architecture should therefore specify what remains possible when connectivity is slow, intermittent, or unavailable.

Human attention is a limited resource. The Robot Revolution Initiative described a broader move toward systems that non-specialists can teach and use across different domains [22]. In crisis operations, this goal is especially demanding because the operator may be supervising several devices while communicating with responders. Simpler programming and control are useful only when the behavior of the robot remains transparent and recoverable.

Studies of telepresence and shared control examine different parts of this problem. Partial and delayed control did not remove the reported sense of embodiment in a humanoid surrogate under the tested conditions [25]. An ontology-based model formalized information needs for situation awareness during shared control [76]. Adaptive levels of automation were proposed for cooperation among a human supervisor, firefighters, and robots [78]. Together, these studies suggest that the operator needs both an understandable representation of the scene and a clear account of which agent currently controls each action.

Telemanipulation and gesture interfaces aim to reduce the effort of issuing commands. An affordance-oriented framework used object recognition and VR support to simplify bimanual tasks with a dual-arm system [79]. A gesture-based interface classified hand postures and actions for multi-robot control [80]. A night-vision surveillance robot represents a simpler remote-control approach based on wireless video [81]. These systems vary greatly in complexity, but all depend on reliable feedback. An intuitive command is unsafe if the operator cannot confirm how the robot interpreted it.

Coordination also consumes information and attention. A self-adaptive model allowed a robot team to form, allocate responsibilities, and reconfigure through interactions among agents in a wildfire scenario [83]. This can reduce the need for a human to assign every local action. However, the system must still reveal why responsibility moved from one robot to another and what happens when the team’s local decisions conflict with the commander’s priorities.

Across the 25 publications assigned to Resource and Knowledge Management, the central lesson is that more data does not automatically produce a better decision. Information must be protected, synchronized, interpretable, and connected to its source and time of acquisition. Communication, semantics, trust, cloud processing, and interface design should therefore be evaluated as parts of the same operational information chain [16,17,18,19,22,25,28,29,30,31,32,33,73,74,75,76,77,78,79,80,81,82,83,84,85].

### 4.4. Robotics Ecosystem and State-of-the-Art Synthesis

The Robotics Ecosystem provides a second view of the same 76 publications. The labels in this perspective overlap and should not be added together. General and Specialized Robots appeared in 47 publications, UAVs in 17, and Technologies and Applications in 45. These counts show that the literature is divided almost equally between physical platforms and the technologies needed to make those platforms useful. They also explain why robot type alone is not an adequate organizing principle.

The General and Specialized Robots category includes mobile rescue platforms, tracked and wheeled robots, continuum robots, legged systems, snake-like and modular designs, soft robots, surveillance units, and multi-purpose prototypes. General mobile bases are attractive because sensors and payloads can be changed between missions. Specialized designs are justified when the environment imposes a clear constraint, such as a narrow void, unstable ground, water, a hazardous atmosphere, or the need to manipulate debris [10,11,23,35,36,39,41,64,65,66,67,68,69].

This variety should not be read as a competition to identify one best platform. It is evidence that crisis environments differ too much for a universal design. A small robot can enter confined spaces but may lack power, communication range, and payload. A larger tracked vehicle can carry sensors and a manipulator but may be unable to pass through a narrow opening. Legged and modular systems gain mobility at the cost of control complexity, while soft mechanisms gain compliance but remain less mature at system level.

The most useful comparison is therefore between mission requirements and platform properties: mobility, size, payload, endurance, sensing, resistance to water and dust, repairability, and the level of control available after communication loss. These properties should be reported under the conditions in which the platform was tested. A robot described as suitable for disaster work after a short indoor demonstration cannot be compared directly with a system tested outdoors, on debris, or during a multi-agency exercise.

The 17 UAV publications emphasize rapid deployment, wide-area coverage, mapping, thermal observation, victim search, and access to locations separated by damaged roads or unstable ground. UAVs are particularly effective during the first stage of an operation, when commanders need an overview and must decide where to send limited response resources [13,14,15,20,53,58,59,85].

Their limitations are equally consistent. Flight time and payload restrict the sensor set, weather affects stability and image quality, and satellite positioning may be unavailable near buildings or indoors. High-rate video and mapping data place demands on the communication link. A UAV that produces an overview but cannot maintain a link or geolocate a finding may create more uncertainty rather than less.

The strongest applications therefore connect aerial observation with another element of the system. A ground robot can inspect a location selected from an aerial map. Fixed sensors can continue monitoring after a UAV leaves. Several micro-aerial vehicles can share viewpoints to build a three-dimensional reconstruction. A UAV can also transport and deploy communication or sensing equipment. In each case, the benefit comes from combining platforms rather than using the aircraft as a stand-alone solution.

The Technologies and Applications category covers simulation, path planning, multi-robot systems, swarm robotics, robot learning, HRI, cameras, LiDAR, gas sensors, WSNs, IoT, communication, data fusion, semantic knowledge, and synchronization. These elements appeared in 45 publications, nearly as often as physical robot platforms. This balance reflects the structure of a real robotic capability: movement without perception, communication, planning, and an operator interface has limited value.

The category also contains the main sources of interdependence. A more accurate perception method may require more computation and energy. A richer map may overload the link. Distributed coordination may remove a central point of failure while making consistency harder to maintain. Cloud processing may provide capacity but increase dependence on external infrastructure. The literature contains promising solutions to each problem, but fewer evaluations of how the solutions behave when combined [12,16,18,21,28,29,30,31,32,33,43,45,46,51,54,55,56,57,60,77].

Table 5 compares the main platform and system classes discussed in the review. The maturity descriptions summarize the type and setting of evidence reported in the selected publications; they are comparative judgments for this review and should not be read as formal Technology Readiness Level assignments.

The maturity labels were assigned from the strongest type of evidence that recurred across the publications in each platform class. Low indicates evidence dominated by simulations, component studies, or isolated prototype demonstrations without repeated task-level evaluation. Moderate indicates repeated prototype or case-oriented evaluations in relevant or semi-realistic settings, but not sustained multi-agency field validation. Low to moderate indicates that working task-level prototypes were reported, while the evidence remained concentrated in short, controlled, or small-scale tests. When evidence differed within a platform class, the label reflects the overall pattern rather than the most advanced individual study. These categories are qualitative comparisons within this review and are not formal Technology Readiness Level assignments.

**Table 5:** Comparison of robotics platforms and supporting system architectures.

Platform or System Class	Main Advantages	Main Limitations	Typical Crisis-Management Tasks	Maturity in the Reviewed Evidence
UAVs [13,14,15,20,53,58,59,85]	Rapid deployment, wide-area coverage, access across damaged or unstable terrain, flexible imaging payloads.	**Limited flight time and payload; sensitivity to weather; weak indoor or near-structure positioning; communication burden.**	Reconnaissance, mapping, thermal observation, victim search, wildfire and landslide assessment, sensor or relay deployment.	Moderate. Repeated prototypes and case-oriented applications were reported, but sustained field validation remained limited.
Wheeled and tracked ground robots [10,23,36,39,40,41,42,47]	Stable close-range inspection, useful payload capacity, prolonged sensing, and the ability to carry manipulators or supplies.	**Rubble, stairs, narrow openings, and unstable surfaces restrict mobility; many systems remain dependent on a communication link.**	Gas and hazard sensing, victim search, local mapping, surveillance, debris inspection, and transport.	Low to moderate. Many working prototypes were evaluated in short or controlled tests.
Specialized robots [11,64,65,66,67,68,69]	Continuum, legged, snake-like, modular, and soft designs can enter confined or irregular spaces and adapt their shape or contact.	**Control complexity, limited payload and endurance, mechanical reliability, and difficult maintenance.**	Void inspection, movement through debris, stability studies, structural reconfiguration, and compliant access.	Low. The evidence was dominated by component studies and prototype demonstrations.
Multi-robot and heterogeneous teams [15,16,17,20,30,31,49,50,51,52,53,54,71,83,85]	Greater coverage, parallel task execution, complementary viewpoints, and partial redundancy when one unit fails.	**Task allocation, collision avoidance, bandwidth demand, synchronization, scaling, and operator workload.**	Distributed search, road clearance, cooperative mapping, formation control, sensor deployment, and combined aerial-ground inspection.	Low to moderate. Simulations and small-team experiments predominated; full-scale exercises were uncommon.
Sensor and communication networks [12,16,17,18,19,20,28,29,30,31,32,33,72,73,74,75,76,77]	Persistent monitoring, communication relays, shared observations, and coordination across mobile and fixed assets.	**Dependence on damaged infrastructure, interoperability and cybersecurity problems, inconsistent or stale data, and coverage gaps.**	Connectivity restoration, environmental monitoring, shared situational awareness, knowledge synchronization, and resource coordination.	Moderate at component level, but lower for complete end-to-end operation under degraded conditions.

The comparison shows why platform choice cannot be separated from mission conditions. UAVs offer speed and coverage, ground robots provide closer and more sustained access, specialized designs address particular mechanical constraints, and multi-robot or networked arrangements combine capabilities. At the same time, every class remains dependent on sensing, communication, energy, and human supervision. Evidence maturity is therefore highest for individual functions and lower for complete systems operating under realistic disruption.

Recent evidence helps distinguish technical novelty from operational maturity. The 2024 account of robot-assisted disaster-response deployments by the German Rescue Robotics Center Task Force [9] provides a current field-based benchmark and shows that deployment logistics, communication, operator preparation, and integration with responders matter alongside platform performance. The reviewed set also contains studies from 2021–2024 on continuum rescue robots, collaborative knowledge systems, multi-quadrotor navigation, landslide monitoring, path planning, and robotic surveillance [11,28,32,53,59,60,61,74]. These publications update the technical picture, but most examine a component, prototype, or bounded scenario rather than a complete multi-agency deployment. Reference [9] is used as contextual field evidence and is not included in the quantitative counts, which remain based on references [10,11,12,13,14,15,16,17,18,19,20,21,22,23,24,25,26,27,28,29,30,31,32,33,34,35,36,37,38,39,40,41,42,43,44,45,46,47,48,49,50,51,52,53,54,55,56,57,58,59,60,61,62,63,64,65,66,67,68,69,70,71,72,73,74,75,76,77,78,79,80,81,82,83,84,85].

**Figure 10:**
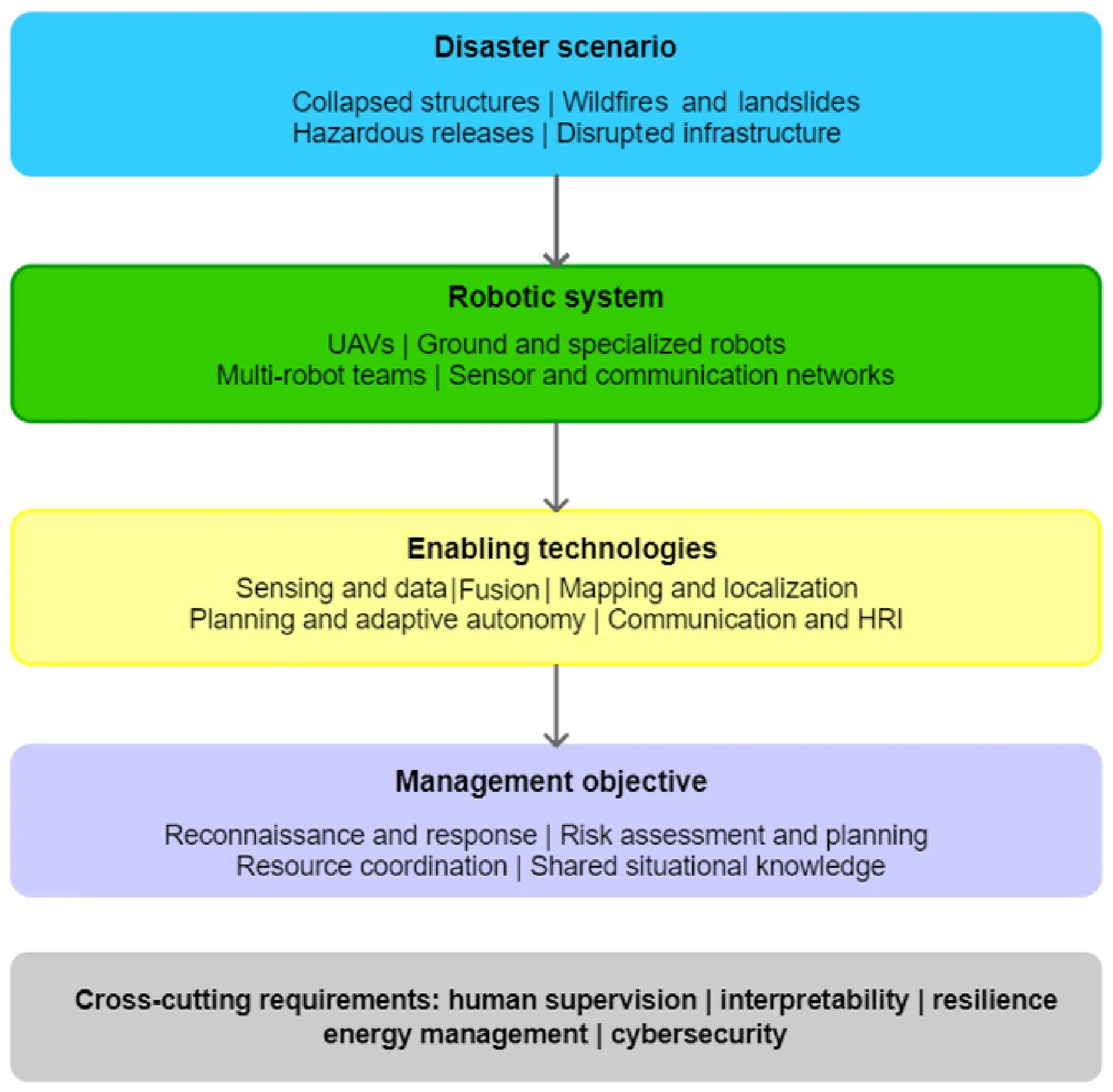
Simplified relationship between disaster conditions, robotic systems, enabling technologies, and management objectives.

Figure 10 reduces the synthesis to four layers: disaster conditions, robotic systems, enabling technologies, and management objectives. The links are many-to-many; the figure is a reading guide, not a fixed operational sequence.

For this reason, technology readiness should be evaluated end to end. The relevant question is not only whether a detector recognizes a victim, but whether the sensor can collect usable data in smoke, the onboard computer can process it in time, the network can transmit the result, the interface shows uncertainty clearly, and the response team can act on the information. A failure at any one of these stages can remove the practical advantage of the robot.

The qualitative evidence helps explain the descriptive pattern in Table 4. Robotics Ecosystem and Research Methodology labels do not form separate groups tied to one management domain. The same platform can support several functions during one mission. A UAV may first provide reconnaissance, then update a risk map, and finally supply data to a shared knowledge system. A ground robot may inspect a hazard, carry a relay, and preserve a route record for later teams. The exploratory *p*-values do not add stronger evidence than this combined descriptive and qualitative interpretation.

The three management domains are therefore useful as analytical viewpoints, but they should not become organizational barriers. Response teams need planning data, planners need reliable observations, and both depend on communication and knowledge management. Research that treats only one layer may still make a valuable technical contribution, but claims of operational readiness should show how that layer connects with the others.

The answer to RQ4 is consequently qualified. The literature demonstrates credible applications in reconnaissance, victim search, environmental sensing, mapping, route planning, task allocation, communication, and shared knowledge. It also shows a transition from isolated remote-controlled devices toward heterogeneous systems that observe, communicate, plan, and coordinate. What remains less developed is evidence that the complete arrangement will continue to work after partial failure, under uncertain information, and with a human operator responsible for consequential decisions.

This distinction between technical capability and operational capability provides the link to Section 5. The limitations identified across the publications do not belong to separate components. Perception uncertainty affects planning; communication loss affects knowledge consistency; computation affects energy; and automation affects operator workload. The next stage of research should therefore evaluate these relationships within complete human–robot systems rather than optimize each component in isolation.

## 5. Discussion

The quantitative results show where the literature is concentrated, while the qualitative review shows what the proposed systems were expected to contribute during an emergency. Considering both views together helps explain why rapid technical development has not yet produced equally strong evidence of field readiness. This section therefore revisits the research questions and then discusses the ten limitations found most often across the 76 publications.

### 5.1. Interpreting the Findings Through the Research Questions

The first four research questions examine different sides of the same development. A larger publication base does not necessarily indicate greater operational readiness, and the frequency of a label does not by itself establish effectiveness in a real incident.

With regard to RQ1, publication activity increased markedly after 2015 within the reviewed sample. The period 2016–2025 contained 61 of the 76 publications, compared with 15 in the preceding decade, and conference papers accounted for 57 records. India was the most frequent country category in the worksheet, with 24 publications. The country distribution did not differ significantly between periods (χ^2^ = 17.37, df = 12, *p* = 0.136). These findings describe the selected publications and should not be generalized to all disaster-robotics research.

RQ2 concerned the thematic and methodological structure of the reviewed literature. Crisis Management and Response was the most frequent management label, appearing in 50 publications, although the qualitative review shows that effective response also depends on planning and information management. Within the Robotics Ecosystem, General and Specialized Robots appeared in 47 publications and Technologies and Applications in 45, while UAVs appeared in 17. Conceptual was the most common methodology label, with 52 publications, followed by Experiment (35) and Case Study (23). Because the coding was multi-label, these figures describe co-occurring features rather than mutually exclusive shares.

For RQ3, the cross-tabulated counts showed no clear one-to-one separation between a Robotics Ecosystem label and the dominant management domain. The displayed exploratory statistics were χ^2^ = 1.72, df = 4, *p* = 0.787 for the Robotics Ecosystem and χ^2^ = 2.82, df = 8, *p* = 0.945 for Research Methodology. These multi-label *p*-values cannot establish independence. Descriptively, General and Specialized Robots appeared less often in Risk Management and Planning, while technologies and methods remained distributed across all three management purposes.

RQ4 helps explain why the absence of a simple association is reasonable. In practice, a rescue robot is part of a wider arrangement involving sensors, planning, communication, data processing, and an operator. A platform may first support reconnaissance, then supply data for a revised plan, and later serve as a node in a shared knowledge network. The three domains organize the review, but these activities remain interdependent during an actual mission.

A *p*-value above 0.05 does not show that periods or categories are equivalent. Interpretation is further limited by 76 studies, unequal period sizes (15 and 61), sparse cells, broad categories, and overlapping labels. Small or context-specific patterns may therefore remain undetected. For this reason, the revised discussion gives priority to the observed counts, proportions, and qualitative evidence and treats the five multi-label statistics as exploratory only.

The qualitative synthesis offers a substantive explanation as well. The same robotic capability often supports several management purposes during one mission, so a strong one-to-one association between technology and domain is not necessarily expected. The tests provide no evidence of a clear separation in this set, but they do not rule out smaller relationships or establish that no relationship exists. The descriptive counts and qualitative findings should therefore be considered together.

### 5.2. Recurrent Limitations and Research Gaps

The fifth research question shifts attention from the proposed solutions to the barriers that restrict their use. Full-text coding identified ten limitations that recurred across platforms, domains, and research methods. The counts in Table 6 are multiple-response frequencies: one publication could contribute to several themes. They indicate the reach of a problem across the reviewed publications, not an equal level of severity in every study.

**Table 6:** Ten most recurrent limitations and research gaps.

No.	Limitation or Gap and Number of Publications	Publications in which the Limitation or Research Gap Was Identified
1	Insufficient validation under real operating conditions—67	[10,11,12,13,14,15,16,17,19,20,21,22,23,24,26,27,30,31,34,35,36,38,39,40,41,42,43,44,45,46,47,48,49,50,51,52,53,54,55,56,57,58,59,60,61,62,63,64,65,66,67,68,70,71,72,73,74,75,76,77,78,79,81,82,83,84,85]
2	Uncertainty in perception, detection, and localization—61	[10,11,12,13,14,15,16,17,21,22,23,25,26,28,29,31,32,34,35,36,37,38,39,40,41,42,43,44,45,46,47,48,49,50,51,52,53,54,55,56,57,58,59,60,61,63,64,66,67,68,69,71,73,74,75,80,81,82,83,84,85]
3	Computational burden and difficulty achieving real-time operation—59	[11,13,14,15,16,17,18,19,21,23,25,26,27,28,30,31,32,33,34,35,36,38,39,41,42,43,45,46,47,48,49,50,51,53,54,55,56,57,58,59,60,61,64,66,67,68,70,71,72,73,74,76,77,78,80,82,83,84,85]
4	Unreliable or limited communication—54	[11,12,13,14,15,16,17,18,19,20,21,22,23,24,25,26,28,29,30,31,33,34,35,36,37,38,39,40,41,44,45,47,48,49,50,51,53,54,57,58,59,66,68,72,73,74,75,77,79,80,81,83,84,85]
5	Limited autonomy and plan adaptation—53	[11,13,14,15,16,21,22,23,25,26,27,28,34,35,36,37,38,39,40,41,42,43,44,45,47,49,50,53,55,57,58,59,60,61,62,63,64,66,68,69,71,72,74,76,77,78,79,80,81,82,83,84,85]
6	Limited reliability and resistance to harsh conditions—53	[11,12,13,14,15,16,17,18,19,20,21,22,23,24,28,29,30,31,33,34,35,36,37,39,40,41,42,43,44,45,47,48,49,50,52,53,54,58,59,60,65,66,67,68,71,73,75,77,81,82,83,84,85]
7	Incomplete integration, interoperability, and trustworthy knowledge flow—52	[12,13,14,15,16,17,18,19,20,21,22,23,26,28,29,30,31,33,34,36,38,40,41,42,43,44,45,46,47,48,52,53,54,55,56,57,59,60,64,66,67,68,70,74,76,77,78,80,81,82,83,84]
8	Scaling and multi-robot coordination problems—47	[11,12,13,14,15,16,17,19,20,21,22,26,27,28,30,31,33,34,35,39,40,42,43,45,47,49,50,51,52,53,54,57,58,60,66,68,70,71,74,75,76,77,78,79,83,84,85]
9	Underdeveloped human–robot collaboration—40	[11,13,14,15,18,19,20,21,22,23,25,26,27,28,29,32,33,35,36,37,39,40,42,47,48,57,62,66,68,69,70,72,74,76,78,79,80,81,82,84]
10	Energy, hardware, and structural constraints—29	[13,14,15,17,19,21,23,33,34,36,37,38,39,40,47,54,57,58,59,63,64,65,66,68,70,73,83,84,85]

#### 5.2.1. Evidence Validation and Operational Robustness

The most frequent weakness was insufficient validation under real operating conditions, identified in 67 publications (Table 6). Simulations, laboratory tests, and short demonstrations are necessary during development, but they do not reproduce unstable debris, water, smoke, weather, radio interference, operator fatigue, and changing priorities at the same time. A method may consequently perform well on a selected metric while the mission as a whole remains fragile [49,53,55,56,57,64,72].

A recent field-based account supports this distinction between a successful component test and operational deployment. The 2024 report from the German Rescue Robotics Center Task Force describes lessons from robot-assisted disaster-response deployments [9]. It is used here as contextual field evidence; it is not part of the 76-publication quantitative dataset and does not change the frequencies reported in Table 6.

This problem is closely connected to limited reliability and resistance to harsh conditions, which appeared in 53 publications (Table 6). Vibration, impact, heat, water, dust, uneven ground, and contamination affect sensors, joints, connectors, and batteries [14,15,16,39,64,67,83]. Future trials should last long enough to reveal battery depletion, sensor drift, synchronization errors, and mechanical wear. Reporting successful runs without failure modes, recovery time, and maintenance requirements provides only a partial picture of system maturity.

#### 5.2.2. Perception, Computation, and Adaptive Autonomy

Uncertainty in perception, detection, and localization appeared in 61 publications (Table 6). Smoke, dust, glare, darkness, occlusion, and irregular geometry reduce the reliability of cameras and localization systems. Thermal imaging, LiDAR, gas sensors, and vision models broaden sensing capacity, but they also fail [10,13,14,21,42,43,45]. A system should therefore report not only an output, but also its confidence, provenance, and known failure conditions. Evaluation should connect false alarms, missed objects, and localization errors with their consequences for the mission.

Computational burden and real-time operation were concerns in 59 publications (Table 6). Three-dimensional mapping, optimization, multi-robot coordination, graph synchronization, and deep learning compete for processor time, memory, bandwidth, and energy [28,32,33,43,46,60,70]. Studies should therefore report end-to-end latency on the target hardware and show how the system safely reduces data quality or update frequency when resources become scarce.

Limited autonomy and plan adaptation were identified in 53 publications (Table 6). Many solutions depended on prior maps, fixed objectives, stable formations, or continuous communication [13,14,35,36,40,71,72]. Under operational conditions, the robot should replan after an obstacle appears, explain why the plan changed, reveal uncertainty, and remain within human-defined safety boundaries. A multi-robot team must also transfer a task when one unit fails or becomes disconnected.

#### 5.2.3. Communication, Interoperability, and Team Coordination

Unreliable or limited communication appeared in 54 publications (Table 6). Debris, distance, damaged infrastructure, spectrum congestion, and platform movement can interrupt a link exactly when it is needed most [12,15,18,19,21,39,81]. A resilient system should prioritize messages, change channels or routes, continue safe local operation, and reconcile data after reconnection. Authentication and key management must also continue to work as team composition and network topology change.

Incomplete integration, interoperability, or trustworthy knowledge flow appeared in 52 publications (Table 6). Components that worked in isolation often used different data formats, coordinate systems, update rates, and security assumptions [16,18,28,29,32,33,77]. Interoperability requires shared descriptions of platform capabilities, tasks, and data provenance, together with interfaces that connect equipment from different vendors. A knowledge system should identify contradictions and stale information rather than merely accumulate more records.

Scaling and multi-robot coordination were problematic in 47 publications (Table 6). A method that works for two or three units may behave differently in a larger and changing team as network traffic, task conflicts, collision risk, and the cost of maintaining a shared world model increase [17,20,30,49,50,52,83]. Evaluation should include temporary disconnections and permanent unit losses. Coordination must be distributed enough to avoid a single point of failure, yet coherent enough to keep the robots’ plans from diverging.

#### 5.2.4. Human Factors and Platform Constraints

Underdeveloped human–robot collaboration was evident in 40 publications (Table 6). Technical studies often reduced the operator’s role to issuing commands, although mission outcomes also depend on recognizing uncertainty, maintaining situation awareness, and taking control without introducing further confusion. Evaluations of VR, tangible tables, gestures, and shared control usually relied on small samples or simplified tasks [21,25,26,27,76,78,79]. Longer studies with responders and commanders should examine cognitive load, trust calibration, missed events, and the quality of control handover.

Energy, hardware, and structural constraints appeared in 29 publications (Table 6). Thermal cameras, LiDAR, manipulators, computers, and radios improve platform capabilities, but also increase mass and energy demand [13,14,36,47,66,73,85]. Planning should cover the entire mission cycle: deployment, rotation, charging or battery replacement, task handover, safe return, and equipment recovery. Modular payloads can reduce unnecessary mass when they are matched to the actual task rather than to the largest possible set of functions.

### 5.3. Priorities for Future Research

The answer to RQ5 is not simply a list of ten separate problems, because the limitations reinforce one another. A weak link reduces the timeliness of the shared operational picture, which in turn degrades route planning and task allocation. Increasing data transmission may improve awareness, but consumes bandwidth, processor time, and energy. A more accurate detector may reduce false alarms but be too slow for the on-board computer [14,21,28,32,33,43,46].

The first priority is to move from component demonstrations to repeatable evaluations of the complete system. Scenarios should be developed with emergency services and combine poor visibility, partial communication loss, platform failure, changing priorities, time pressure, and energy constraints. Reporting should cover not only the final outcome, but also mission time, detection performance, false alarms, localization error, communication availability, energy consumption, recovery time, and operator workload [22,44,45,46,55,56,57,61,64,70,71,72].

The second priority is resilience to partial degradation. The system should retain safe local functions after losing a link, replan locally, transfer tasks, and synchronize knowledge when communication returns. This requires perception, autonomy, communication, cybersecurity, and energy management to be investigated together rather than optimized as isolated layers [13,14,18,19,28,29,30,31,32,33,51,52,53,73,83].

The third priority concerns people and interoperability. Shared descriptions of platform capabilities, tasks, and data provenance should make it easier to connect equipment from different vendors with existing emergency-service systems. At the same time, studies must establish whether the operator understands the automation, can judge its confidence appropriately, and can take control without creating further confusion [16,17,18,20,21,22,25,26,27,28,29,30,32,33,48,59,76,77,78,79,80,84]. Only this combined approach can show whether a solution has moved beyond the prototype stage.

This review has several limitations. Scopus and Web of Science were searched during the same search period using the same conceptual strategy, thematic keyword groups, publication-year range, and English-language restriction. The database-specific syntax differed because the platforms use different field tags and commands. The retained Scopus notes document 115, 110, and 101 records at successive stages. The exact text originally entered in WoS was not retained, so the equivalent WoS expression was rebuilt from the documented concepts, limits, keyword groups, and retained export. That search produced 117 records, of which 29 remained after refinement.

The 101 Scopus and 29 WoS records formed a combined set of 130. Nineteen records occurred in both databases, and one duplicate was present within Scopus. Removing these 20 duplicates left 110 unique records; 76 were included and 34 were not included. The retained files do not contain a separate reason code for every non-included record, so unavailable full texts cannot be separated reliably from full-text eligibility exclusions.

The country categories follow the retained worksheet, but the original rule for selecting among multiple affiliations was not preserved. The classification used overlapping labels, and no independent second coding record is available for calculating inter-coder agreement. In addition, sparse cells and multi-label coding limit the interpretation of the chi-square results. These statistics are therefore presented as exploratory summaries, while the conclusions rely mainly on descriptive counts and qualitative evidence. The findings apply to the reviewed sample and should not be read as a complete estimate of the maturity of the entire field.

## 6. Conclusions

Taken together, the 76 publications show rapid but uneven development within the reviewed literature. The studies describe a wider range of robotic tasks than the earlier literature, but a successful prototype test does not by itself demonstrate dependable performance during an emergency. The five research questions help separate areas with a substantial body of work from areas where evidence remains limited.

The clearest change in this sample concerns publication volume. Sixty-one studies appeared in 2016–2025, and conference papers accounted for 57 of the 76 records. India was the most frequent country category in the retained worksheet. However, the country comparison did not identify a statistically significant difference between the two periods. The increase in publications should therefore not be interpreted as evidence that long-term, comparable field validation has advanced at the same rate.

The observed thematic distribution was broadly similar across the two periods, although sparse categories limit statistical interpretation. Crisis Management and Response appeared most often, while General and Specialized Robots was the leading platform category. Technologies and Applications covered a large share of the supporting solutions, and conceptual research predominated methodologically. These categories are not mutually exclusive. One system may combine a specialized robot, a UAV, sensors, planning, and several forms of validation, so the totals should not be read as numbers of separate studies.

Within this sample, the descriptive evidence does not support a simple assignment of one technology type to one management domain. The Robotics Ecosystem value (χ^2^ = 1.72, df = 4, *p* = 0.787) and Research Methodology value (χ^2^ = 2.82, df = 8, *p* = 0.945) are exploratory summaries of overlapping labels. They are not evidence of independence. In an operational setting, the same platform may support reconnaissance, planning, and knowledge sharing during one mission.

The practical value of robots is most evident when they reduce human exposure and shorten the time needed for reconnaissance. They can support mapping, communication, task allocation, and information exchange [12,13,14,15,16,17,18,19,20,34,35,36,37,38,39,40,41,42,43,44,45,46,47,48,49,50,51,52,53]. Their usefulness, however, does not arise from mobility alone. It also depends on perception quality, route safety, link stability, interface clarity, and the ability to incorporate data into an existing decision process [21,26,27,28,29,30,31,32,33,54,55,56,57,58,59,60,61,62,63,64,65,66,67,68,69,70,71,72,73,74,75,76,77,78,79,80,81,82,83,84,85].

The largest gap concerns the resilience of the complete system, not the next isolated algorithm. Publications repeatedly reported insufficient field validation, uncertainty in perception and localization, computational burden, unreliable communication, limited autonomy, reliability problems, and incomplete integration. Trials should therefore be longer, reproducible, and conducted under deliberately degraded conditions. They should cover the full human–robot system and use a shared set of measures: mission time, detection performance, localization error, link availability, energy use, recovery time, and operator workload.

The broad search vocabulary did not lead to an equally strong presence of every topic in the final set. Industrial robots appeared mainly in studies of automation, transportation, and competence development [22,63]. Nanorobots, agricultural and social robots, and robot-assisted surgery did not form distinct streams among the 76 publications. This resulted from the final relevance assessment, not from the omission of those terms from the search queries.

The goal, then, is not autonomy at any cost. In crisis operations, greater value lies in a system that can continue safely after a partial failure, communicates its limitations clearly, and leaves critical decisions under human control. Reaching that level of readiness requires platforms, sensors, planning, communication, knowledge management, and operator oversight to be designed together. Their performance as one integrated system should be treated as the measure of operational maturity.

The classifications were assigned by the authors and were not independently double-coded; consequently, the category-based findings should be interpreted with appropriate caution. Future updates of the review should include independent coding of at least a subset of the publications and report inter-coder agreement.

## Data Availability

The publication-level coding used for the recurrent-limitations analysis is provided in Appendix B (Table A1), and the publications assigned to each limitation are listed in Table 6. Aggregated data supporting the remaining tables and figures are contained in the article. The complete manually coded worksheet used for the remaining descriptive classifications is not publicly available.

## Abbreviations

The following abbreviations are used in this manuscript:

AHP	Analytic Hierarchy Process
ARES	Advanced Robot for Extraction and Survey
AWARE	project integrating UAVs, ground cameras, and wireless sensors
CNN	Convolutional Neural Network
DOI	Digital Object Identifier
DRASB	Disaster Response and Surveillance Bot
GIS	Geographic Information System
GPS	Global Positioning System
HFKN	Hastily Formed Knowledge Network
HRI	Human–Robot Interaction
IoT	Internet of Things
LiDAR	Light Detection and Ranging
NFV	Network Function Virtualization
RDF	Resource Description Framework
RGB-D	red–green–blue imaging with depth information
RGS⊕	RDF graph-synchronization system described in two reviewed publications [28,32]
RQ	Research Question
RTMA	Robot and Task Mean Allocation
SDN	Software-Defined Networking
SENEKA	Situation Responsive Networking of Mobile Robots for Disaster Management
SLAM	Simultaneous Localization and Mapping
UAS	Unmanned Aircraft System
UAV	Unmanned Aerial Vehicle
UGV	Unmanned Ground Vehicle
VIEW-FINDER	robotic data-acquisition and integration project for crisis management
VR	Virtual Reality
WoS	Web of Science
WSN	Wireless Sensor Network

## Appendix A. Database Search Strategies

Scopus and Web of Science were searched during the same search period using the same search logic, thematic concept groups, publication-year range, and language restriction. The complete Scopus query was retained and is reproduced below. Because the databases use different field tags and syntax, an equivalent WoS expression was required. Its exact original text was not saved, so the WoS syntax shown below was rebuilt from the retained export and documented search conditions. This reconstruction concerns the database-specific expression, not the scope or timing of the search.

Scopus, recovered query: TITLE-ABS (“Crisis Management” OR “Disaster Management” AND “Robots”) AND PUBYEAR > 2005 AND PUBYEAR < 2026 AND (EXCLUDE (DOCTYPE, “le”)) AND (EXCLUDE (SUB-JAREA, “ARTS”) OR EXCLUDE (SUBJAREA, “PSYC”) OR EXCLUDE (SUBJAREA, “BIOC”) OR EXCLUDE (SUBJAREA, “CHEM”) OR EXCLUDE (SUBJAREA, “CENG”) OR EXCLUDE (SUBJAREA, “ECON”) OR EXCLUDE (SUBJAREA, “SOCI”)) AND (LIMIT-TO (LANGUAGE, “English”)) AND (LIMIT-TO (EXACTKEY-WORD, “Disaster Management”) OR LIMIT-TO (EXACTKEYWORD, “Crisis Management”) OR LIMIT-TO (EX-ACTKEYWORD, “Human Resource Management”) OR LIMIT-TO (EXACTKEYWORD, “Risk Management”) OR LIMIT-TO (EXACTKEYWORD, “Environmental Management”) OR LIMIT-TO (EXACTKEYWORD, “Information Management”) OR LIMIT-TO (EXACTKEYWORD, “Search And Rescue”) OR LIMIT-TO (EXACTKEY-WORD, “Simulation”) OR LIMIT-TO (EXACTKEYWORD, “Situation Awareness”) OR LIMIT-TO (EXACTKEY-WORD, “Distributed Knowledge”) OR LIMIT-TO (EXACTKEYWORD, “Motion Planning”)) AND (LIMIT-TO (EXACTKEYWORD, “Robots”) OR LIMIT-TO (EXACTKEYWORD, “Robotics”) OR LIMIT-TO (EXACTKEY-WORD, “Intelligent Robots”) OR LIMIT-TO (EXACTKEYWORD, “Multipurpose Robots”) OR LIMIT-TO (EX-ACTKEYWORD, “Industrial Robots”) OR LIMIT-TO (EXACTKEYWORD, “Robot Programming”) OR LIMIT-TO (EXACTKEYWORD, “Multi-robot Systems”) OR LIMIT-TO (EXACTKEYWORD, “Swarm Robotics”) OR LIMIT-TO (EXACTKEYWORD, “Robot”) OR LIMIT-TO (EXACTKEYWORD, “Rescue Robot”) OR LIMIT-TO (EXACT-KEYWORD, “Unmanned Aerial Vehicles (uav)”) OR LIMIT-TO (EXACTKEYWORD, “Drones”) OR LIMIT-TO (EXACTKEYWORD, “Unmanned Aerial Vehicle”) OR LIMIT-TO (EXACTKEYWORD, “Sensors”) OR LIMIT-TO (EXACTKEYWORD, “Unmanned Aerial Vehicles”) OR LIMIT-TO (EXACTKEYWORD, “Aerial Vehicle”) OR LIMIT-TO (EXACTKEYWORD, “Nanorobots”) OR LIMIT-TO (EXACTKEYWORD, “Mobile Robot”) OR LIMIT-TO (EXACTKEYWORD, “Mobile Robots”) OR LIMIT-TO (EXACTKEYWORD, “Robot Applications”) OR LIMIT-TO (EXACTKEYWORD, “Robot Learning”) OR LIMIT-TO (EXACTKEYWORD, “Human Robot Interaction”) OR LIM-IT-TO (EXACTKEYWORD, “Internet Of Things”) OR LIMIT-TO (EXACTKEYWORD, “Search And Rescue Robot”) OR LIMIT-TO (EXACTKEYWORD, “Social Robots”) OR LIMIT-TO (EXACTKEYWORD, “Agricultural Robots”) OR LIMIT-TO (EXACTKEYWORD, “Simulation”) OR LIMIT-TO (EXACTKEYWORD, “Cameras”) OR LIMIT-TO (EXACTKEYWORD, “Wireless Sensor Networks”) OR LIMIT-TO (EXACTKEYWORD, “Visually Guided Robots”) OR LIMIT-TO (EXACTKEYWORD, “Robot Assisted Surgery”) OR LIMIT-TO (EXACTKEYWORD, “Rescue Oper-ations”)).

WoS search rebuilt from the retained documentation: TS = ((“Crisis Management” OR “Disaster Management”) AND Robots) AND PY = (2006–2025) AND LA = (English). This search returned 117 records.

WoS management refinement terms: Disaster Management; Crisis Management; Human Resource Management; Risk Management; Environmental Management; Information Management; Search And Rescue; Simulation; Situation Awareness; Distributed Knowledge; Motion Planning.

WoS robotics and technology refinement terms: Robots; Robotics; Intelligent Robots; Multipurpose Robots; Industrial Robots; Robot Programming; Multi-robot Systems; Swarm Robotics; Robot; Rescue Robot; Unmanned Aerial Vehicles (UAV); Drones; Unmanned Aerial Vehicle; Sensors; Unmanned Aerial Vehicles; Aerial Vehicle; Nanorobots; Mobile Robot; Mobile Robots; Robot Applications; Robot Learning; Human Robot Interaction; Internet of Things; Search and Rescue Robot; Social Robots; Agricultural Robots; Simulation; Cameras; Wireless Sensor Networks; Visually Guided Robots; Robot Assisted Surgery; Rescue Operations. Applying the documented refinement left 29 records. The exact original WoS refinement expression cannot be recovered from the retained files.

Complete reconstructed WoS expression: #1 TS = ((“Crisis Management” OR “Disaster Management”) AND Ro-bots) AND PY = (2006–2025) AND LA = (English); #2 AK = (“Disaster Management” OR “Crisis Management” OR “Human Resource Management” OR “Risk Management” OR “Environmental Management” OR “Information Management” OR “Search And Rescue” OR Simulation OR “Situation Awareness” OR “Distributed Knowledge” OR “Motion Planning”); #3 AK = (Robots OR Robotics OR “Intelligent Robots” OR “Multipurpose Robots” OR “Industrial Robots” OR “Robot Programming” OR “Multi-robot Systems” OR “Swarm Robotics” OR Robot OR “Rescue Robot” OR “Unmanned Aerial Vehicles (UAV)” OR Drones OR “Unmanned Aerial Vehicle” OR Sensors OR “Unmanned Aerial Vehicles” OR “Aerial Vehicle” OR Nanorobots OR “Mobile Robot” OR “Mobile Robots” OR “Robot Applications” OR “Robot Learning” OR “Human Robot Interaction” OR “Internet of Things” OR “Search and Rescue Robot” OR “Social Robots” OR “Agricultural Robots” OR Simulation OR Cameras OR “Wireless Sensor Networks” OR “Visually Guided Robots” OR “Robot Assisted Surgery” OR “Rescue Operations”); #4 #1 AND #2 AND #3. This combined syntax is a transparent reconstruction of the documented refinement conditions, not the verbatim command originally entered in WoS. The retained notes record 117 results for the base search and 29 records after refinement.

## Appendix B. Publication-by-Limitation Coding Matrix

Table A1 lists the final limitation codes assigned to every included publication. L1-L10 correspond to the ten categories defined in Section 2.5 and listed in Table 6. A blank code would mean that none of the ten recurrent categories was assigned.

**Table A1:** Final limitation codes assigned to included publications.

Reference	Assigned Limitation Codes	Reference	Assigned Limitation Codes
[10]	L1, L2	[48]	L1, L2, L3, L4, L6, L7, L9
[11]	L1, L2, L3, L4, L5, L6, L8, L9	[49]	L1, L2, L3, L4, L5, L6, L8
[12]	L1, L2, L4, L6, L7, L8	[50]	L1, L2, L3, L4, L5, L6, L8
[13]	L1, L2, L3, L4, L5, L6, L7, L8, L9, L10	[51]	L1, L2, L3, L4, L8
[14]	L1, L2, L3, L4, L5, L6, L7, L8, L9, L10	[52]	L1, L2, L6, L7, L8
[15]	L1, L2, L3, L4, L5, L6, L7, L8, L9, L10	[53]	L1, L2, L3, L4, L5, L6, L7, L8
[16]	L1, L2, L3, L4, L5, L6, L7, L8	[54]	L1, L2, L3, L4, L6, L7, L8, L10
[17]	L1, L2, L3, L4, L6, L7, L8, L10	[55]	L1, L2, L3, L5, L7
[18]	L3, L4, L6, L7, L9	[56]	L1, L2, L3, L7
[19]	L1, L3, L4, L6, L7, L8, L9, L10	[57]	L1, L2, L3, L4, L5, L7, L8, L9, L10
[20]	L1, L4, L6, L7, L8, L9	[58]	L1, L2, L3, L4, L5, L6, L8, L10
[21]	L1, L2, L3, L4, L5, L6, L7, L8, L9, L10	[59]	L1, L2, L3, L4, L5, L6, L7, L10
[22]	L1, L2, L4, L5, L6, L7, L8, L9	[60]	L1, L2, L3, L5, L6, L7, L8
[23]	L1, L2, L3, L4, L5, L6, L7, L9, L10	[61]	L1, L2, L3, L5
[24]	L1, L4, L6	[62]	L1, L5, L9
[25]	L2, L3, L4, L5, L9	[63]	L1, L2, L5, L10
[26]	L1, L2, L3, L4, L5, L7, L8, L9	[64]	L1, L2, L3, L5, L7, L10
[27]	L1, L3, L5, L8, L9	[65]	L1, L6, L10
[28]	L2, L3, L4, L5, L6, L7, L8, L9	[66]	L1, L2, L3, L4, L5, L6, L7, L8, L9, L10
[29]	L2, L4, L6, L7, L9	[67]	L1, L2, L3, L6, L7
[30]	L1, L3, L4, L6, L7, L8	[68]	L1, L2, L3, L4, L5, L6, L7, L8, L9, L10
[31]	L1, L2, L3, L4, L6, L7, L8	[69]	L2, L5, L9
[32]	L2, L3, L9	[70]	L1, L3, L7, L8, L9, L10
[33]	L3, L4, L6, L7, L8, L9, L10	[71]	L1, L2, L3, L5, L6, L8
[34]	L1, L2, L3, L4, L5, L6, L7, L8, L10	[72]	L1, L3, L4, L5, L9
[35]	L1, L2, L3, L4, L5, L6, L8, L9	[73]	L1, L2, L3, L4, L6, L10
[36]	L1, L2, L3, L4, L5, L6, L7, L9, L10	[74]	L1, L2, L3, L4, L5, L7, L8, L9
[37]	L2, L4, L5, L6, L9, L10	[75]	L1, L2, L4, L6, L8
[38]	L1, L2, L3, L4, L5, L7, L10	[76]	L1, L3, L5, L7, L8, L9
[39]	L1, L2, L3, L4, L5, L6, L8, L9, L10	[77]	L1, L3, L4, L5, L6, L7, L8
[40]	L1, L2, L4, L5, L6, L7, L8, L9, L10	[78]	L1, L3, L5, L7, L8, L9
[41]	L1, L2, L3, L4, L5, L6, L7	[79]	L1, L4, L5, L8, L9
[42]	L1, L2, L3, L5, L6, L7, L8, L9	[80]	L2, L3, L4, L5, L7, L9
[43]	L1, L2, L3, L5, L6, L7, L8	[81]	L1, L2, L4, L5, L6, L7, L9
[44]	L1, L2, L4, L5, L6, L7	[82]	L1, L2, L3, L5, L6, L7, L9
[45]	L1, L2, L3, L4, L5, L6, L7, L8	[83]	L1, L2, L3, L4, L5, L6, L7, L8, L10
[46]	L1, L2, L3, L7	[84]	L1, L2, L3, L4, L5, L6, L7, L8, L9, L10
[47]	L1, L2, L3, L4, L5, L6, L7, L8, L9, L10	[85]	L1, L2, L3, L4, L5, L6, L8, L10

## References

[1] Wilk-Jakubowski G., Harabin R., Ivanov S. (2022). Robotics in crisis management: A review. Technol. Soc..

[2] Altay N., Green W.G. (2006). OR/MS Research in Disaster Operations Management. Eur. J. Oper. Res..

[3] Pescaroli G., Alexander D. (2018). Understanding Compound, Interconnected, Interacting, and Cascading Risks: A Holistic Framework. Risk Anal..

[4] Balcik B., Beamon B.M., Krejci C.C., Muramatsu K.M., Ramirez M. (2010). Coordination in Humanitarian Relief Chains: Practices, Challenges and Opportunities. Int. J. Prod. Econ..

[5] Murphy R.R., Stover S. (2008). Rescue Robots for Mudslides: A Descriptive Study of the 2005 La Conchita Mudslide Response. J. Field Robot..

[6] Kruijff G.J.M., Kruijff-Korbayová I., Keshavdas S., Larochelle B., Janíček M., Colas F., Liu M., Pomerleau F., Siegwart R., Neerincx M.A. (2014). Designing, Developing, and Deploying Systems to Support Human-Robot Teams in Disaster Response. Adv. Robot..

[7] Delmerico J., Mintchev S., Giusti A., Gromov B., Melo K., Horvat T., Cadena C., Hutter M., Ijspeert A., Floreano D. (2019). The Current State and Future Outlook of Rescue Robotics. J. Field Robot..

[8] Erdelj M., Król M., Natalizio E. (2017). Wireless Sensor Networks and Multi-UAV Systems for Natural Disaster Management. Comput. Netw..

[9] Surmann H., Daun K., Schnaubelt M., von Stryk O., Patchou M., Böcker S., Wietfeld C., Quenzel J., Schleich D., Behnke S. (2024). Lessons from Robot-Assisted Disaster Response Deployments by the German Rescue Robotics Center Task Force. J. Field Robot..

[10] Das T., Sut D.J., Gupta V., Gohain L., Kakoty P., Kakoty N.M. (2020). A Mobile Robot for Hazardous Gas Sensing.

[11] Yamauchi Y., Ambe Y., Nagano H., Konyo M., Bando Y., Ito E., Arnold S., Yamazaki K., Itoyama K., Okatani T. (2022). Development of a Continuum Robot Enhanced with Distributed Sensors for Search and Rescue. Robomech J..

[12] Kuntze H.-B., Frey C., Emter T., Petereit J., Tchouchenkov I., Müller T., Tittel M., Worst R., Pfeiffer K., Walter M. (2014). Situation Responsive Networking of Mobile Robots for Disaster Management.

[13] Lee S., Har D., Kum D. (2017). Drone-Assisted Disaster Management: Finding Victims via Infrared Camera and Lidar Sensor Fusion.

[14] Calderon D., Cordero R., Gonzalez A., Lemus A., Fajardo J. (2021). Galileo Aid Drone: A System Integration for Autonomous Wildfire Assistants.

[15] Fernando E., Dampage U., Hanees A., Dissanayake N., Karunasiri D., Abeysinghe K. (2018). Integrated Rescue Group for Landslide Disaster Management.

[16] Sardouk A., Mansouri M., Merghem-Boulahia L., Gaïti D., Rahim-Amoud R. (2010). Multi-Agent System Based Wireless Sensor Network for Crisis Management.

[17] Seminara G., Fontanelli D. (2018). First Responders Robotic Network for Disaster Management.

[18] Mouradian C., Jahromi N.T., Roch R.H. (2018). NFV and SDN-Based Distributed IoT Gateway for Large-Scale Disaster Management. IEEE Internet Things J..

[19] Jawhar I., Mohamed N., Al-Jaroodi J. (2020). Secure Communication in Multi-Robot Systems.

[20] Maza I., Caballero F., Capitan J., Martinez-De-Dios J.R., Ollero A. (2011). A Distributed Architecture for a Robotic Platform with Aerial Sensor Transportation and Self-Deployment Capabilities. J. Field Robot..

[21] Stotko P., Krumpen S., Schwarz M., Lenz C., Behnke S., Klein R., Weinmann M. (2019). A VR System for Immersive Teleoperation and Live Exploration with a Mobile Robot.

[22] Sziebig G., Korondi P. (2015). Effect of Robot Revolution Initiative in Europe—Cooperation Possibilities for Japan and Europe. FAC-PapersOnLine.

[23] Pal S., Malarvizhi S. (2021). Smart Robot Design for Terrain Traverse.

[24] Kaji A.H., Bair A., Okuda Y., Kobayashi L., Khare R., Vozenilek J. (2008). Defining Systems Expertise: Effective Simulation at the Organizational Level—Implications for Patient Safety, Disaster Surge Capacity, and Facilitating the Systems Interface. Acad. Emerg. Med..

[25] Aymerich-Franch L., Petit D., Ganesh G., Kheddar A. (2016). Embodiment of a Humanoid Robot Is Preserved During Partial and Delayed Control.

[26] Gertman D.I., Bruemmer D.J., Hartley R.S. (2007). Improving Emergency Response and Human-Robotic Performance.

[27] Merrad W., Héloir A., Kolski C., Krüger A. (2022). RFID-Based Tangible and Touch Tabletop for Dual Reality in Crisis Management Context. J. Multimodal User Interfaces.

[28] Berger C., Doherty P., Rudol P., Wzorek M. (2023). RGS⊕: RDF Graph Synchronization for Collaborative Robotics. Auton. Agents Multi-Agent Syst..

[29] Doherty P., Berger C., Rudol P., Wzorek M. (2021). Hastily Formed Knowledge Networks and Distributed Situation Awareness for Collaborative Robotics. Auton. Intell. Syst..

[30] Choudhury S., Dey S., Mukherjee A. (2019). A Distributed Semantic Knowledge Framework for Collaborative Robotics.

[31] Capitán J., Merino L., Caballero F., Ollero A. (2011). Decentralized Delayed-State Information Filter (DDSIF): A New Approach for Cooperative Decentralized Tracking. Robot. Auton. Syst..

[32] Berger C., Doherty P., Rudol P., Wzorek M. (2024). A Summary of the RGS⊕ System: An RDF Graph Synchronization System for Collaborative Robotics.

[33] Thakur A., Sahoo S., Mukherjee A., Halder R. (2023). Making Robotic Swarms Trustful: A Blockchain-Based Perspective. J. Comput. Inf. Sci. Eng..

[34] AlAli Z.T., Alabady S.A. (2024). Techniques and Methods for Managing Disasters and Critical Situations. Nat. Hazards.

[35] Kostavelis I., Gasteratos A. (2017). Robots in Crisis Management: A Survey.

[36] Joshi A., Nagarjun C.S., Srinivas R. (2017). The DRASB—Disaster Response and Surveillance Bot.

[37] Joseph J., Berchmans N., Varghese M.R., Krishnan M., Arya B.S., Antony M. (2019). Arduino Based Automatic Human Seeker Robot for Disaster Management.

[38] Vashistha S., Shruti, Khatri Y. (2019). A.H.R.D-Autonomous Human Recognition Device.

[39] Chari K.S., Ranjan R., Baig M.N.A., Naresh A. (2017). Mobile Controlled Multipurpose Rescue Robot Using DTMF Technology.

[40] Narayana Raju P.J., Gaurav Pampana V., Pandalaneni V., Gugapriya G., Baskar C. (2022). Design and Implementation of a Rescue and Surveillance Robot Using Cross-Platform Application.

[41] Giri R., Ramkumar G., Kirthana L. (2021). ARES-Advanced Robot for Extraction and Survey.

[42] De Cubber G., Doroftei D. (2010). Human Victim Detection and Stereo-Based Terrain Traversability Analysis for Behaviour-Based Robot Navigation. Using Robots in Hazardous Environments: Landmine Detection, De-Mining and Other Applications.

[43] Baudoin Y., Doroftei D., De Cubber G., Berrabah S.A., Pinzon C., Warlet F., Gancet J., Motard E., Ilzkovitz M., Nalpantidis L. (2009). View-Finder: Robotics Assistance to Fire-Fighting Services and Crisis Management.

[44] Ellore V., Anand L.N., Tripathi S. (2020). Image Deblurring and Localization for Disaster Response and Humanitarian Assistance. Procedia Comput. Sci..

[45] Li L., Ota K., Dong M., Borjigin W. (2017). Eyes in the Dark: Distributed Scene Understanding for Disaster Management. IEEE Trans. Parallel Distrib. Syst..

[46] Yajima Y., Kim S., Chen J., Cho Y.K. Fast Online Incremental Segmentation of 3D Point Clouds from Disaster Sites. Proceedings of the 38th ISARC.

[47] Fattah S.A., Chowdhury D., Haider M.Z., Sarkar M., Refat M., Rabbi G., Masud S.B., Shahnaz C. (2016). Dynamic Map Generating Rescuer Offering Surveillance Robotic System with Autonomous Path Feedback Capability.

[48] Nosirov K., Begmatov S., Arabboev M. (2020). Analog Sensing and Leap Motion Integrated Remote Controller for Search and Rescue Robot System.

[49] Nath A., Arun A.R., Niyogi R. (2019). A Distributed Approach for Road Clearance with Multi-Robot in Urban Search and Rescue Environment. Int. J. Intell. Robot. Appl..

[50] Abraham L., Biju S., Biju F., Jose J., Kalantri R., Rajguru S. (2019). Swarm Robotics in Disaster Management.

[51] Din A., Jabeen M., Zia K., Khalid A., Saini D.K. (2018). Behavior-Based Swarm Robotic Search and Rescue Using Fuzzy Controller. Comput. Electr. Eng..

[52] Yinka-Banjo C.O., Osunmakinde I.O., Bagula A. (2017). Robustness of Cooperative Behaviour Model on N Robot-Based Multi-Robot Systems: Application to Mine Emergency and Disaster Management.

[53] Dsouza J.M., Rafikh R.M., Nair V.G. (2023). Autonomous Navigation System for Multi-Quadrotor Coordination and Human Detection in Search and Rescue. J. Robot. Mechatron..

[54] Alarabi S., Lei T., Santora M., Luo C., Sellers T. (2024). Multi-Robot Path Planning Using Potential Field-Based Simulated Annealing Approach.

[55] Bertolino M., Tanzi T.J. (2020). Towards 3d Simulation for Disaster Intervention Robot Behaviour Assessment. Adv. Radio Sci..

[56] Tanzi T., Bertolino M. (2020). 3D Simulation to Validate Autonomous Intervention Systems Architecture for Disaster Management.

[57] Khalyeyev D., Hnětynka P., Bureš T. (2018). A Virtual Playground for Testing Smart Cyber-Physical Systems.

[58] Agarwal A., Shukla V., Singh R., Gehlot A., Garg V. (2018). Design and Development of Air and Water Pollution Quality Monitoring Using Iot and Quadcopter.

[59] Reynoso Vanderhorst H.D. Unmanned Aerial System Integration for Monitoring and Management of Landslide: A Case of Dominican Republic. Proceedings of the 38th ISARC.

[60] Thakre N.K., Nimma D., Turukmane A.V., Singh A.K., Rohatgi D., Bangaru B. (2024). Dynamic Path Planning for Autonomous Robots in Forest Fire Scenarios Using Hybrid Deep Reinforcement Learning and Particle Swarm Optimization. Int. J. Adv. Comput. Sci. Appl..

[61] Orisatoki M.O., Amouzadi M., Dizqah A.M. (2024). A Heuristic Informative-Path-Planning Algorithm to Map Unknown Areas and A Benchmark Solution.

[62] Subash T.D., Basheer N., Sreelakshmi K.S., Jayaprakash A., Mathai L. (2020). Autonomous Public Transportation System. Mater. Today Proc..

[63] Abdullah-Al-Kaiser M., Paul D.J., Khan A.I., Shahnaz C., Fattah S.A. (2017). A Cost Effective GPS Guided Autonomous Object Transporter Robot for Disaster Management and Industrial Automation.

[64] Dan A., Saha S.K., Krishna K.R. (2024). A Review on the Stability of Biped Robots. J. Indian Inst. Sci..

[65] Takishima Y., Yoshida K., Khosla A., Kawakami M., Furukawa H. (2021). Fully 3D-Printed Hydrogel Actuator for Jellyfish Soft Robots. ECS J. Solid State Sci. Technol..

[66] Reddy C.S.S., Patlolla S., Agrawal A., Anupama K.R. (2017). SQ-BOT—A Modular Robot Prototype for Self-Reconfiguring Structures.

[67] Siva I., Sangaran D.K., Praveen Balan P.R., Rajesh Kanna R., Siddharth P., Sankar I. (2020). Modal Analysis on the Additive Manufactured Robotic Segments: Effect of Printing Densities on the Vibrational Properties. Mater. Today Proc..

[68] Kulkarni A.V., Chawla R. (2007). Structure and Analysis of a Snake-Like Robot.

[69] Abhimanyu, Rane T., Godiyal R., Reddy S.C.S. (2019). 3DoBot—A Modular Robot for Wheel and Chain Coordinate Structures.

[70] Cheikhrouhou O., Koubâa A., Zaard A. (2016). Analytical Hierarchy Process Based Multi-Objective Multiple Traveling Salesman Problem.

[71] Yu S., Barca J.C. (2015). Autonomous Formation Selection for Ground Moving Multi-Robot Systems.

[72] Pralet C., Lesire C. (2014). Deployment of Mobile Wireless Sensor Networks for Crisis Management: A Constraint-Based Local Search Approach.

[73] Sharevski F. (2018). Leveraging Cellular Internet-of-Things for Resilient and Robust Disaster Management.

[74] Hansen G., Benson J.R., Danish M.R., Truong K., Yu X., Saniie J. (2024). Advanced Robotic Surveillance for Urban Air Quality Safety.

[75] Abbas M.Z., Abu Baker K., Ayaz M., Mohamed H., Tariq M., Ahmed A., Faheem M. (2018). Key Factors Involved in Pipeline Monitoring Techniques Using Robots and WSNs: Comprehensive Survey. J. Pipeline Syst. Eng. Pract..

[76] Smets N.J.J.M., Neerincx M.A., Jonker C.M., Båberg F. (2017). Ontology-Based Situation Awareness Support for Shared Control: Extended Abstract.

[77] Mouradian C., Yangui S., Glitho R.H. (2018). Robots As-a-Service in Cloud Computing: Search and Rescue in Large-Scale Disasters Case Study.

[78] Habib L., Pacaux-Lemoine M.-P., Millot P. (2017). Adaptation of the Level of Automation According to the Type of Cooperative Partner.

[79] Gorjup G., Dwivedi A., Elangovan N., Liarokapis M. (2019). An Intuitive, Affordances Oriented Telemanipulation Framework for a Dual Robot Arm Hand System: On the Execution of Bimanual Tasks.

[80] Tornow M., Al-Hamadi A., Borrmann V. (2013). Gestic-Based Human Machine Interface for Robot Control.

[81] Sharma D., Chauhan U. (2020). War Spying Robot with Wireless Night Vision Camera.

[82] Watremez X., Marblé A., Baron T., Marcarian X., Dubucq D., Donnat L., Cazes L., Foucher P.-Y., Danno R., Elie D. (2018). Remote Sensing Technologies for Detecting, Visualizing and Quantifying Gas Leaks.

[83] Georgé J.-P., Gleizes M.-P., Garijo F.J., Noël V., Arcangeli J.-P. (2010). Self-Adaptive Coordination for Robot Teams Accomplishing Critical Activities.

[84] Kumar Y.B., Ilaiyaraja S. (2015). Smart Interface for Sensors in Robot.

[85] Padilla M.L.F., Chirarattananon P., Bandala A.A., Vicerra R.R.P., Baldovino R.G., Dadios E.P. (2019). Formation-Based 3D Mapping of Micro Aerial Vehicles.

